# Effect of 9,10-Dimethyl-1,2-Benzanthracene on the Mouse Ovary. Ovarian Tumorigenesis

**DOI:** 10.1038/bjc.1970.20

**Published:** 1970-03

**Authors:** T. Krarup

## Abstract

**Images:**


### EFFECT OF 9,10-DIMETHYL-1,2-BENZANTHRACENE ON THE MOUSE OVARY. OVARIAN TUMORIGENESIS

T. KRARUP

*From the Finsen Laboratory, The Finsen Institute, Copenhagen, Denmark*

Received for publication September 11, 1969

**SUMMARY.—Groups of immature and mature mice were treated once with DMBA by oral or intraperitoneal route, and the subsequent bilateral sequence of ovarian changes leading to the development of unilateral granulosa cell tumour was studied.**

**Early post-treatment changes included disappearance of oocytes and follicles as well as increase of the stroma mass. The neoplastic development was closely correlated to the rate of oocyte disappearance. The faster oocytes were eliminated, the earlier tumours appeared. The early post-treatment changes led to a stage of potential preneoplasia, characterized by diffuse luteinization of the ovarian parenchyma. In some preneoplastic ovaries the luteinized tissue underwent neoplastic transformation and developed into invasive luteoma. In other preneoplastic ovaries foci of granulosa-like tumour cells appeared in the luteinized tissue, representing the stage of microscopic granulosa cell tumour. However, such microtumours could also develop within pre-existing luteomata. Autoradiography after injection of thymidine-$^3$H suggested that the granulosa-like tumour cells developed as the result of undifferentiated proliferation of luteinized cells.**

**So far the pathological ovarian evolution occurred bilaterally as well as unilaterally. However, when a microscopic granulosa cell tumour by further growth became a macroscopic granulosa cell tumour the contralateral ovary invariably atrophied. This ultimate unilateral development coincided with a continuous production of oestrogen by the granulosa cell tumour. The reason for the contralateral atrophy is discussed in relation to the influence of the hormonal balance on ovarian tumorigenesis.**

THE carcinogenic hydrocarbon 9,10-dimethyl-1,2-benzanthracene (DMBA) administered to sensitive strains of mice affects the ovaries and induces granulosa cell tumours regardless of whether it is applied externally to the skin (Howell *et al.*, 1954; Marchant, 1957; Mody, 1960), orally (Biancifiori *et al.*, 1961; Jull *et al.*, 1966; Krarup, 1967; Kuwahara, 1967), intraperitoneally (Krarup, 1967; Kuwahara, 1967), intravenously (Kuwahara, 1967), subcutaneously (Shisa and Nishizuka, 1968), or directly to the surface of the ovary (Krarup, 1969*a*).

Disappearance of oocytes precedes the development of ovarian tumours induced by X-irradiation (Guthrie, 1958), intrasplenic ovary transplantation (Guthrie, 1957), and DMBA treatment (Marchant, 1957; Krarup, 1967). A common mechanism in the experimental induction of ovarian tumours in mice would therefore seem to be the elimination of ova (Jull *et al.*, 1966; Marchant, 1967; Krarup, 1969*a*).

Marchant (1961*b*) applied the two-stage model of initiation and promotion in experimental carcinogenesis (Berenblum and Shubik, 1947) to the chemical induction of ovarian tumours in mice and suggested DMBA to be the initiation factor and the pituitary influence the promoting factor. This was based on the observation, that preneoplastic changes (including depletion of oocytes and follicles) readily developed after DMBA application in hypophysectomized animals, while the further tumour development only occurred in the presence of the pituitary (Marchant, 1961*a*).

The initiation phase has recently been described in detail (Krarup, 1969*b*). DMBA immediately affects the small oocytes and destroys a large proportion of them. Secondarily the developing follicles are reduced in number and the ovaries are prematurely depleted of oocytes.

The promotion phase is known to depend on the pituitary function (Marchant, 1961*a*); however, the presence of a normal ovary with its hormone production inhibited tumorigenesis in unilateral preneoplastic ovaries grafted from donors pretreated with DMBA (Marchant, 1960). The promotion phase therefore seems to include a hormone dependent stage. As oocyte destruction occurs synchronously and to the same extent in the two ovaries of a mouse after DMBA treatment (Krarup, 1969*b*), it would be expected that also tumour promotion occurred equally in the two ovaries. However, the ultimate ovarian tumours are unilateral (Howell *et al.*, 1954; Mody, 1960; Krarup, 1967; Kuwahara, 1967; Shisa and Nishizuka, 1968).

In the present study the gradual development of pathological changes in both ovaries after DMBA treatment is described, and the correlation to the rate of oocyte disappearance (Krarup, 1969*a*) is further explored. The histogenic pathways of tumour development are studied, and a stage is described in the neoplastic process, after which only one ovary continues its development while the other one is arrested and atrophies. This stage coincides with changes in the hormonal balance.

### MATERIALS AND METHODS

Four hundred and ninety-one female virgin mice of the Bagg strain were used for the investigation (Table I). The experimental procedure and information on the strain and care of animals, as well as on the histological technique, has previously been reported together with total and differential oocyte counts (Krarup, 1969*b*).

*Control groups.*—Two different groups of mice served as controls.

(1) 63 untreated virgin mice
(2) 30 immature mice were treated orally with 0·05 ml. olive oil and 39 intraperitoneally with 0·25 ml. olive oil.

The control mice were killed and examined at different ages (Table I, a–c).

*DMBA orally.*—DMBA dissolved in olive oil was fed through a thin gastric tube to anaesthetized mice. One hundred and eleven animals were treated at the age of 21 days, *i.e.* in immaturity. Ninety-nine of these received 0·25 mg. DMBA and 12 received 0·5 mg. DMBA. Sixty-one mice were treated at the age of 4 months, *i.e.* in maturity, with 1 mg. DMBA.

In order to describe the proliferation pattern of ovarian tissue after exposure to the carcinogen 10 mice aged 6–9 months were injected intraperitoneally with 100 $\mu$Ci tritiated thymidine ($^3$HTdR). The mice were killed 1 hour later and

TABLE 1.—*Number and Age of Mice in Control and Experimental Groups*

| Age at death | *a* Untreated controls (total 63 mice) | | *b* Olive oil orally at 21 days (total 30 mice) | | *c* Olive oil intraperitoneally at 21 days (total 39 mice) | | *d* DMBA orally at 21 days (total 111 mice) | | *e* DMBA orally at 4 months (total 61 mice) | | *f* DMBA intraperitoneally at 21 days (total 122 mice) | | *g* DMBA intraperitoneally at 4 months (total 65 mice) | |
|---|---|---|---|---|---|---|---|---|---|---|---|---|---|---|
| | Killed (63) | Found dead (0) | Killed (29) | Found dead (1) | Killed (37) | Found dead (2) | Killed (98) | Found dead (13) | Killed (49) | Found dead (12) | Killed (78) | Found dead (44) | Killed (36) | Found dead (29) |
| (days) | | | | | | | | | | | | | | |
| 22 | 4 | — | — | — | — | — | 4 | 1 | — | — | 3 | — | — | — |
| 28 | 3 | — | — | — | — | — | 4 | 1 | — | — | 4 | 1 | — | — |
| 35 | 3 | — | — | — | — | — | 6 | 1 | — | — | 4 | 5 | — | — |
| 42 | 3 | — | — | — | — | — | 5 | — | — | — | 2 | 5 | — | — |
| 49 | 6 | — | — | — | — | — | 6 | — | — | — | 3 | 3 | — | — |
| months | | | | | | | | | | | | | | |
| 3 | 4 | — | — | — | — | — | 11 | — | — | — | 11 | 3 | — | — |
| 4 | — | — | — | — | — | — | — | — | — | — | — | 2 | — | — |
| 4½ | — | — | — | — | — | — | — | — | 3 | — | — | 2 | 3 | 1 |
| 5 | — | — | — | — | — | — | — | — | 4 | — | — | 3 | 4 | 5 |
| 6 | 12 | — | 9 | — | 14 | 2 | 27* | 1 | 4 | — | 28 | 4 | 10 | 8 |
| 7 | — | — | — | — | — | — | — | — | — | — | — | 8 | — | 3 |
| 8 | — | — | — | — | — | — | — | 2 | — | 3 | — | — | — | 1 |
| 9 | 11 | — | 11 | — | 11 | — | 19† | 4 | 11 | 1 | 16 | 4 | 8 | 1 |
| 10 | — | — | — | — | — | — | — | — | — | — | — | 1 | — | 3 |
| 11 | — | — | — | 1 | — | — | — | 3 | — | 3 | — | 1 | — | 1 |
| 12 | 13 | — | 9 | — | 12 | — | 16 | — | 17 | 1 | 7 | 2 | 8 | 2 |
| 13 | — | — | — | — | — | — | — | — | — | 1 | — | — | — | 1 |
| 14 | — | — | — | — | — | — | — | — | | 1 | — | — | — | 2 |
| 15 | 4 | — | — | — | — | — | — | — | 10 | 2 | — | — | 3 | 1 |

* 3 mice and † 7 mice received 100 $\mu$Ci $^{3}$HTdR, and autoradiographs were prepared.

autoradiographs of the serially sectioned ovaries were prepared (Pedersen and Krarup, 1969) alternating with simple histological sections.

*DMBA intraperitoneally.*—One hundred and twenty-two immature mice aged 21 days were injected intraperitoneally with 0·25 mg. DMBA dissolved in olive oil. Sixty-five mature mice aged 4 months were treated intraperitoneally with 1 mg. DMBA in olive oil.

The DMBA treated mice were killed or died spontaneously at different ages (Table I, *d–g*). Ovaries and organs macroscopically abnormal were removed for examination.

*Vaginal smears.*—In order to obtain information on the hormonal status of the animals daily vaginal smears were taken from 55 control mice and 151 experimental mice for 3 weeks before scheduled sacrifice.

### RESULTS

### *Ovaries*

#### *Control mice*

The ovaries of all control mice were macroscopically normal with recognizable follicles and corpora lutea. Spontaneous ovarian tumours did not occur.

The microscopic appearance of the normal Street mouse ovary at different ages has previously been reported (Krarup, 1969*a*). The ovarian morphology in Bagg mice is similar and is therefore only summarized here. Pathological changes were not found at any age.

At all ages oocytes in different stages of follicle development characterized the organs. Degeneration of follicles was likewise observed at all ages, most markedly in the juvenile period. The stroma appeared in increasing amounts after the fifth week of life and seemed to be derived from degenerated follicular material; it could be slightly luteinized in areas. After the age of 42 days corpora lutea were a characteristic structure in all ovaries. With increasing age pigmented cells (Fekete, 1946; Deane and Fawsett, 1952; Thung *et al.*, 1956) appeared in areas of luteinized stroma as well as in corpora lutea, and in old animals pigmented tissue became a prominent structure. Empty rings and pseudofollicles (" anovular follicles ") (Thung *et al.*, 1956; Krarup, 1967) as well as " Sertoli bodies " (Engle, 1946; Thung *et al.*, 1956) were occasionally found in the ovaries of old animals. They were spherical and composed of cells with bright nuclei containing a dark-staining nucleolus and abundant cytoplasm with a reticular structure (Fig. 15).

The typical senile ovary had few oocytes, follicles, and corpora lutea. The main component was non-luteinized stroma with many pigmented cells.

#### *DMBA treated mice*

A total of 359 mice was treated with DMBA; 98 of these were found dead before scheduled killing (see Table I). Because of sometimes advanced autolysis, microscopic examination of ovaries and other organs was impossible.

Two hundred and sixty-one DMBA treated animals were killed at different ages according to a predetermined schedule.

A macroscopic ovarian tumour was found in a number of animals. With one exception they were all unilateral, the contralateral ovary usually being considerably smaller than normal (" pinhead ovary "). The largest diameter of the tumours varied from 3–5 mm. to 30 mm. with 10–20 mm. as the most common size. Large subcapsular haemorrhages and necrotic areas containing cell detritus were present in the large tumours. They were usually well encapsuled, though adhesions to and displacement of neighbouring organs were common, sometimes causing hydronephrosis and renal infection. All the ovaries in animals without a macroscopic tumour were of normal size or only slightly smaller than normal.

*Microscopic examination.*—Histological evaluation made it possible to divide the ovaries into two groups: (1) Those which had not developed tumours (non-tumour group) but showed (*a*) early post-treatment changes, (*b*) potential pre-neoplastic changes (Krarup, 1969*a*), (*c*) premature senility or (*d*) atrophy, and (2) those which had developed tumours (tumour group). According to the different stages of neoplastic development, these were described as (*a*) luteoma, (*b*) microscopic granulosa cell tumour (Mic. G.) or macroscopic granulosa cell tumour (Mac. G.). This classification, however, is arbitrary, and transitional stages were seen. Histologically normal ovaries were never found among the DMBA treated animals.

In the following the main characteristics of each type of pathology is described. Their occurrences at different ages appear from Fig. 21 and 22, which summarize the bilateral ovarian histology of all experimental mice advanced further than early post treatment changes.

1. *Non-tumour group*

(*a*) *Early post-treatment changes.*—A reduction in the oocyte number occurred immediately after DMBA application, most markedly in the intraperitoneally treated animals (Krarup, 1969*b*). In the first week after feeding DMBA to 21-days-old mice, degenerated small oocytes and empty spaces left by them were noted in the ovarian cortex. The number of developing follicles was reduced 2 weeks after treatment, and follicle degeneration and the amount of stroma increased concurrently. Some empty rings and pseudofollicles, and a rim of small dark-staining cells appeared in the periphery in the third week. Though the oocyte population at the beginning of the fourth post-treatment week (age 42 days) was reduced to 10% of a normal complement, follicles came to ovulation, eggs were seen in the tube and corpora lutea were formed.

Similar changes were seen after feeding DMBA to 4-month-old animals. In addition a luteinization of the stroma and an accumulation of corpora lutea occurred. In spite of the severe reduction of the number of germ cells, ovulation continued.

After intraperitoneal injection of DMBA to immature mice the capillaries were dilated and an extensive degeneration of developing follicles occurred already in the first week. Empty rings appeared earlier than after treatment by mouth. The ovaries looked " underdeveloped " because of the severe degenerative changes, the low number of healthy follicles and the lack of corpora lutea, which were still not found 4 weeks after treatment. Degenerative changes could proceed to leave only traces of atrophied follicular material or patches of connective tissue at the site of the ovaries (total degeneration).

Such severe ovarian degeneration also happened after intraperitoneal injection to 4-month-old mice; however, most of them showed luteinization of stroma as well as persistence and merging of corpora lutea, thus tending towards potential preneoplasia.

(*b*) *Potential preneoplasia* was the result of further progression of the early post-treatment changes. In orally treated animals potential preneoplasia (preneoplastic ovaries) was characterized by a diffuse luteinization of the stroma and an accumulation of corpora lutea (Fig. 1) which merged and formed a central mass of diffusely luteinized material together with the luteinized stroma (Fig. 2). This centre was covered by a more or less complete rim of empty rings and pseudofollicles (Fig. 2 and 3). Recently ruptured follicles and ova in the tube were seen at times, even when the preneoplastic changes were advanced and only a few oocytes were left in the ovaries (Fig. 4). The degree of preneoplastic development was correlated to the number of oocytes present in the ovaries. At comparable ages, the less advanced preneoplasia was found in those ovaries that contained the highest number of oocytes. Survival of oocytes thus seemed to delay the process.

The prenoeplastic ovaries appeared somewhat differently in the intraperitoneally treated animals. The ovaries were considerably smaller than after feeding of DMBA, and empty rings or pseudofollicles were few or absent. Dilatation of vessels was common and luteinized tissue was the main structure found. In only a few of these ovaries could remnants of corpora lutea be identified.

In preneoplastic ovaries as well as in other types of pathological ovaries " Sertoli bodies " were at times seen (Fig. 15).

(*c*) *Premature senile changes* were occasionally found. Such ovaries were like the senile ovaries in old control mice, but they occurred at younger ages. Non-luteinized stroma and pigmented tissue were the main components.

(*d*) *Atrophy*.—The atrophic ovaries were very small (pinhead ovaries). A broad rim of empty rings, pseudofollicles and small dark-staining cells covered a centre of stroma cells and pigmented cells (Fig. 5). Luteinized tissue was not found in the atrophic ovaries.

2. *Tumour group*

(*a*) *Luteoma*.—The ovaries of this group were of normal or slightly reduced size. On microscopical examination all of the structures described for potential preneoplastic ovaries could be found, however, the reason for classifying them as luteoma was that the luteinized tissue showed neoplastic properties: penetration through the ovarian capsule, invasion into the periovarian fat (Fig. 6 and Fig. 11), and areas of mitotic activity within the luteinized tissue. The cells of the luteoma showed nuclear anisocytosis and the abundant cytoplasma contained eosinophilic granula. The luteomas contained very few (6–9-month-old animals) or no oocytes (9–12-month-old animals).

In one case a papilliferous cystadenoma was present within an invasive luteoma (Fig. 6).

(*b*) *Microscopic granulosa cell tumour* (*Mic. G.*).—Mic. G. were characterized by proliferation of granulosa-like tissue within an ovary that was macroscopically not enlarged. Such development was seen either in potential preneoplastic ovaries (early occurrence of Mic. G.) or in luteomas (late occurrence of Mic. G.).

The tumour cells were microscopically similar to granulosa cells of normal follicles. They were found in small or large foci without any specific pattern, and numerous mitosis suggested a rapid growth. Small cysts were at times present. In typical cases of Mic. G. developing within a preneoplastic ovary, large tumour foci reduced the remaining luteinized tissue to a semilunar shell at one of the poles of the ovary (Fig. 7 and 8), or a preneoplastic ovary-rest could be found as an appendix to the tumour (Fig. 9). Sometimes areas of theca cell proliferation were found side by side with the granulosa-like tumour cells. One or two oocytes were occasionally found in the preneoplastic ovary-rest (Fig. 9), but usually oocytes or follicles were absent in ovaries showing Mic. G. As in preneoplastic ovaries the abnormal ovarian growth seemed to be correlated to the rate of disappearance of oocytes: at the age of 3 months, 2 mice had a Mic. G.; these ovaries contained considerably fewer oocytes than others, that still showed preneoplastic changes.

When Mic. G. developed within a luteoma (Fig. 10) the proliferation of granulosa-like cells could be found in areas of invasion (Fig. 11) or diffusely among the luteoma cells (Fig. 12). In other ovaries foci had grown to a bulk of granulosa-like tumour tissue replacing most of the previously luteinized organ (Fig. 13).

---

### EXPLANATION OF PLATES

FIG. 1.—Preneoplastic ovary showing accumulation and merging of corpora lutea. The section contains a residual follicle. × 34.

FIG. 2.—Preneoplastic ovary. The centre of diffusely luteinized cell material is covered by a rim of empty rings and pseudofollicles. × 34.

FIG. 3.—Preneoplastic ovary with a peripheral rim of empty rings and pseudofollicles and a centre of luteinized tissue. The frame refers to Fig. 18. × 85.

FIG. 4.—Preneoplastic luteinized ovary showing signs of ovulation with two eggs in the tube and two recently ruptured follicles. Only eight oocytes were left in this ovary. × 20.

FIG. 5.—Atrophic ovary composed of a broad rim of empty rings and pseudofollicles and a centre of stroma and pigmented cells. × 37.

FIG. 6.—Luteoma. The luteoma cells invade the periovarian fat capsule. A papilliferous cystadenoma has developed within the luteoma. × 92.

FIG. 7.—Early microscopic granulosa cell tumour arising in a preneoplastic ovary. Small cysts are present within foci of granulosa-like tumour cells. Preneoplastic tissue is present at the upper pole of the ovary. × 37.

FIG. 8.—Microscopic granulosa cell tumour (detail of Fig. 7). The granulosa-like tissue, showing mitosis, has reduced preneoplastic luteinized tissue to a semilunar shell. × 230.

FIG. 9.—Early microscopic granulosa cell tumour arising in a preneoplastic ovary. The upper (larger) mass consists of granulosa-like tissue without any specific growth pattern apart from the presence of a cyst; the lower mass shows a more whirled structure and is composed mainly of theca-like cells. A preneoplastic ovary rest containing two follicles is found as an appendix to the tumour. × 23.

FIG. 10.—Late microscopic granulosa cell tumour developing within a luteoma. Luteoma cells invade the periovarian fat at the hilus. In this area as well as in central islands of luteoma tissue granulosa-like cells arise diffusely. × 23.

FIG. 11.—Granulosa-like cells (darkstaining) arising among luteoma cells in area of invasion (detail of Fig. 10). × 92.

FIG. 12.—Granulosa-like cells (darkstaining) arising diffusely in islands of luteoma tissue (detail of Fig. 10). × 92.

FIG. 13.—A microscopic granulosa cell tumour has grown to a bulk of tumour tissue. A triangle shaped area of luteinized tissue is still recognizable in the centre of the tumour. × 37.

FIG. 14.—Transitional stage between microscopic and macroscopic granulosa cell tumour. Cysts are present and the formation of follicle-like structures is found. × 23.

FIG. 15.—Small focus of granulosa-like cells centrally in a luteinized preneoplastic ovary (intraperitoneal DMBA). A " Sertoli body " is seen at the periphery (arrow). × 92.

FIG. 16.—Macroscopic granulosa cell tumour showing highly differentiated growth with formation of follicle-like structures. × 92.

FIG. 17.—Stroma cell tumour composed of stroma-like cells arranged in a pseudofollicular pattern. × 92.

FIG. 18.—Autoradiogram of preneoplastic ovary prepared of a section 5 $\mu$ from Fig. 3. A focus of luteinized tissue contains many labelled cells while adjacent areas of luteinized tissue have not incorporated the label. × 230.

FIG. 19.—Autoradiogram of a luteoma showing labelled cells in zone of invasion. × 370.

FIG. 20.—Autoradiogram of a macroscopic granulosa cell tumour with a follicular pattern showing many labelled cells. × 370.

Krarup

5

6

7

8

9

10

11

12

Krarup.

Krarup.

17

18

19

20

Krarup.

When this progressed further a more specific pattern of tumour growth including the formation of follicle-like structures could be found (Fig. 14).

The Mic. G. developing after intraperitoneal injection of DMBA appeared somewhat differently. The tumour cells arose in the luteinized preneoplastic ovaries in small foci (Fig. 15) or diffusely in larger areas.

(c) *Macroscopic granulosa cell tumour* (*Mac. G.*).—These tumours were macroscopically recognizable and were composed predominantly of granulosa-like cells.

The histological pattern varied with the size; the smaller tumours were solid without any particular arrangement of their cells while the large tumours were highly differentiated showing follicle-like structures (Fig. 16) and Call-Exner bodies. Haemorrhages, necrotic areas, and fibrous scars—sometimes with cartilage formation and ossification—were seen. Parts of the tumours could consist of pigmented tissue or stroma-like cells. No mitotic figures were seen in these parts of the tumour while they were numerous in the granulosa-like tissue. Oocytes, normal follicles or luteinized tissue were not observed.

A few tumours were composed exclusively of stroma-like cells arranged in pseudofollicles similar to those seen in preneoplastic ovaries (Fig. 17). Such " stroma cell tumours " were thought to represent a growth type different from the Mac. G.

*Proliferation pattern of pathological ovarian tissue*

Ten mice, fed with DMBA in immaturity, were injected intraperitoneally with 100 $\mu$Ci $^3$HTdR when aged 6 to 9 months and killed 1 hour later. Autoradiographs were prepared of their ovaries (Table I, *d*). These 20 ovaries represented various stages of the neoplastic ovarian development, which made it possible to study the incorporation pattern of the labelled DNA-precursor into different types of pathological ovarian tissue.

The few follicles present in *potential preneoplastic ovaries* showed a labelling index of the granulosa cells comparable to that of normal mice (Pedersen and Krarup, 1969). Some empty rings and pseudofollicles had a few labelled cells, but the majority of them were unlabelled. In the diffusely luteinized central tissue none or very few cells had incorporated the label. Pigmented tissue was unlabelled.

In some of the preneoplastic ovaries, however, luteinized areas or whole " corpora " of luteinized cells (Fig. 3) had a high percentage of labelled cells suggesting an active cell proliferation (Fig. 18). This was thought to be the first stage of neoplastic proliferation recognized in the DMBA treated ovaries.

In the *luteomas* a few cells became labelled and the labelling index was rather low. However, in zones of invasion into the periovarian fat many cells incorporated the label (Fig. 19).

Foci of Mic. G. within a preneoplastic ovary or a luteoma had a high percentage of labelled cells, while the remaining parts of the organ were labelled as described above.

Many cells of the Mac. G. incorporated $^3$HTdR. Within the same tumour, however, areas of low or no labelling as well as areas of high labelling were found. Especially areas with a follicular pattern had many labelled cells (Fig. 20). Some parts of the tumours thus seemed to proliferate faster than others. Areas within the tumours composed of stroma-like cells or pigmented tissue never contained labelled cells.

Labelled cells were only very rarely found in atrophic ovaries.

*Incidence of ovarian tumours*

The total incidence of animals with ovarian tumours, as well as the relative incidence of the tumour types found in the different experimental groups at various ages, are recorded in Fig. 21 and 22. Because of the small number of mice killed at old age after intraperitoneal injection of DMBA in maturity—a result of a high early mortality (Table I, *g*)—the tumour incidences in this group are inconclusive. However, some trends may be deduced from the results of the other groups. All mice finally developed tumours, but there was a difference in the incidence of the different tumour types as well as in the speed, with which tumours developed.

Luteoma was mainly seen in animals treated orally in immaturity. At 6 months the total tumour incidences after oral and intraperitoneal DMBA treatment in immaturity were 13/27 (48 %) and 19/28 (68 %) respectively. This was due to an early development of Mic. G. in the intraperitoneally treated animals, while most ovaries in the oral group at this age were still preneoplastic. At 9 months many of the preneoplastic ovaries had developed further to luteoma or Mic. G. and the total tumour incidence became comparable to that in the intraperitoneally treated mice of the same age. However, in these animals the relative incidence of Mac. G. was now considerably higher than among orally treated mice. It would thus appear that tumour development occurred faster after intraperitoneal injection than after oral ingestion.

Comparing mice treated orally at 21 days and at 4 months of age, it is seen that at 9 months the tumour incidences were comparable. In other words, the latency period was considerably shorter when DMBA was administered to mature animals.

*Vaginal smears*

Vaginal smears were taken for 3 weeks on 55 control mice and 151 DMBA treated animals at different ages before killing.

The smears were classified according to the degree of oestrogen stimulation:

(1) Anoestrus (AOE), *i.e.* no oestrogen stimulation. These smears consisted only of leucocytes, degenerated cells and mucus.

(2) Regular oestrus cycles (ROE) of 4 to 5 days' duration suggesting a regular, cyclic oestrogen stimulation.

(3) Irregular oestrus cycles (IOE) suggesting an irregular oestrogen stimulation. Mixed smears of high cornification (cornified cells mixed with leucocytes, nucleated cells and mucus cells) for 1 to 4 days' duration alternated with mixed smears of low or no cornification for durations of up to 10 days.

(4) Permanent oestrus (POE) suggesting a continuous oestrogen stimulation of the vagina. The smears showed cornified cells only or cornified cells predominantly mixed with leucocytes, nucleated cells or mucus cells.

Almost all of the control mice showed ROE-smears. The vaginal smear pattern of the DMBA treated mice has been correlated with the pathology of their ovaries (Table II); (as in Fig. 21 and 22 the mice have been classified according to the more advanced of the two ovaries).

It appears that mice with early post treatment changes or potential preneoplastic changes or luteoma usually showed ROE- or IOE-smears suggesting a cyclic—though often irregular—oestrogen stimulation.

Half of the mice bearing a Mic. G. had ROE-, IOE-, or AOE-smears, while in

TABLE II.—*Vaginal Smears in Control and DMBA Treated Mice in Relation to the Bilateral Ovarian Histology. Mice Showing Different Stages of Neoplastic Development in the Two Ovaries are Classified by the More Advanced Ovary (Capitals).*

| | Number of mice with: | | | | |
|---|---|---|---|---|---|
| | No oestrogen stimulation | Cyclic oestrogen stimulation | | Continuous oestrogen stimulation | |
| Type of ovarian histology | AOE | ROE | IOE | IOE→POE | POE |
| CONTROL OVARIES (total 55 mice) | 2 | 53 | — | — | — |
| EARLY POST TREATMENT CHANGES (total 16 mice) | — | 3 | 13 | — | — |
| POTENTIAL PRENEOPLASIA (total 19 mice) | | | | | |
| potential preneoplasia (19 mice) | 1 | 9 | 8 | — | 1 |
| LUTEOMA (total 7 mice) | | | | | |
| luteoma (3 mice) | 2 | — | — | — | 1 |
| potential preneoplasia (3 mice) | 1 | — | 2 | — | — |
| atrophy (1 mouse) | — | — | 1 | — | — |
| MIC. G. CELL TUMOUR (total 50 mice) | | | | | |
| Mic. G. cell tumour (13 mice) | 2 | 1 | 3 | 2 | 5 |
| luteoma (11 mice) | 4 | — | 3 | — | 4 |
| potential preneoplasia (13 mice) | 1 | 3 | 6 | 1 | 2 |
| senile (1 mouse) | — | — | — | 1 | — |
| atrophy (12 mice) | 2 | — | — | 1 | 9 |
| MAC. G. CELL TUMOUR (total 56 mice) | | | | | |
| stroma cell tumour (2 mice) | — | — | — | — | 2 |
| Mac. G. cell tumour (4 mice) | — | — | 1 | — | 3 |
| luteoma (1 mouse) | — | — | — | — | 1 |
| potential preneoplasia (1 mouse) | — | — | 1 | | — |
| atrophy (48 mice) | 4 | 1 | 1 | — | 42 |

the other half IOE-smears followed by POE- or pure POE-smears were seen. This was particularly marked when the ovary opposite to a Mic. G. was atrophic.

In the experimental mice so far mentioned regular, irregular or continuous oestrogen stimulation occurred independent of whether a few oocytes were left in the ovaries or total depletion of oocytes had already been accomplished (Parkes, 1926; Zylicz *et al.*, 1967).

Forty-eight mice out of 56 with unilateral Mac. G. showed POE-smears suggesting a permanent oestrogen production by these tumours.

One mouse with bilateral " stroma cell tumour " and 2 mice with bilaterally atrophied ovaries (not tabulated) had AOE-smears, *i.e.* no oestrogen stimulation.

*Lesions Other Than Ovarian Tumours and Survival of Animals*

Lesions other than ovarian tumours among control and experimental mice are listed on Table III.

Pulmonary tumours were often seen and usually multiple. Their morphology concurred with that described by Murphy (1966). They occurred spontaneously among ageing control mice; DMBA, however, markedly enhanced their development.

Leukaemia—except for a single case among the control mice (not listed)—only occurred among DMBA treated mice. The disease was often generalized including liver, spleen, thymus and lymph nodes, but isolated cases of thymic enlargement occurred. This could be marked and cause the death of the mouse.

TABLE III.—*Lesions Other Than Ovarian Tumours Occurring Among Control Mice (a, b, c) as well as After Feeding of DMBA in Immaturity (d) or in Maturity (e), and After Intraperitoneal Injection of DMBA in Immaturity (f) or in Maturity (g). (Small Letters Refer to Those of Table I)*

| Age at autopsy (months) | Number of mice with different lesions/total number of mice in each age group | | | | | | | | | | | | | | | | | | | | | | | | | | |
|---|---|---|---|---|---|---|---|---|---|---|---|---|---|---|---|---|---|---|---|---|---|---|---|---|---|---|---|
| | Pulmonary tumours | | | | | | | Leukaemia | | | | Uterine excrescences | | | | Ascites | | | | Peritoneal adhesions | | | | Miscellaneous | | | |
| | a | b | c | d | e | f | g | d | e | f | g | d | e | f | g | d | e | f | g | d | e | f | g | d | e | f | g |
| <3 | — | — | — | — | — | — | — | — | — | 1/44 | — | — | — | 1/44 | — | — | — | — | — | — | — | — | — | — | — | — | — |
| 4–6 | — | — | — | 5/28 | — | 5/39 | — | 2/28 | — | 7/39 | — | 1/28 | — | 11/39 | — | — | — | 3/39 | 14/31 | — | — | 1/39 | 10/31 | 1/28* | — | — | — |
| 7–9 | 2/11 | — | 2/11 | 12/25 | 5/15 | 9/28 | 1/13 | — | 2/15 | 5/28 | 1/13 | — | — | 6/28 | 2/13 | — | — | 1/28 | 5/13 | — | — | — | 3/13 | 3/25† | — | 1/28‡ | — |
| 10–12 | 1/13 | 1/9 | 1/12 | 16/19 | 12/21 | 9/11 | 2/14 | 2/19 | 1/21 | 1/11 | 1/14 | — | — | 8/11 | 2/14 | — | — | — | 1/14 | — | — | 1/11 | 1/14 | — | 1/21* | 1/11† | — |
| 13–15 | 1/4 | — | — | — | 4/14 | — | 2/7 | — | 1/14 | — | — | — | — | — | — | — | — | — | 2/7 | — | — | — | — | — | — | — | 1/7† |

* mammary tumour; † hepatoma; ‡ haemangioma on the back and in the liver.

Excrescences on the uterine horns were almost exclusively observed after intraperitoneal injection of DMBA. They appeared as multiple small vesicles on the uterine surface. Microscopically they were thin-walled cysts containing lymphocytes or cell free fluid. As the deeper layers of the uterine wall showed marked dilatation of lymph capillaries they probably represented cystic protrusions belonging to the lymph system.

Ascites—usually milky—and peritoneal fibrinous adhesions were only seen in intraperitoneally treated animals.

The incidence of mammary tumour was very low.

Death occurred in all groups of treated animals throughout the experiment. (The figures are listed in Table I.) The frequent early death after intraperitoneal injection has been noted previously (Kuwahara, 1967); it was due to peritonitis or a general toxic action of the carcinogen. At times death was due to haemoperitoneum from ruptured ovarian tumours, or to pulmonary tumours, thymic enlargement, or ileus.

Macroscopic ovarian tumours were frequently seen in mice that died spontaneously. Seven out of 10 mice and 11 out of 20 mice that had received oral and intraperitoneal treatment in immaturity respectively and that died after the age of 6 months, had unilateral macroscopic tumours. Among animals treated in immaturity by oral and intraperitoneal route, 9 out of 12 mice and 4 out of 12 mice respectively which died after the age of 8 months had a macroscopic ovarian tumour.

### DISCUSSION

### *Disappearance of oocytes and its relation to tumorigenesis*

The reduction of the number of oocytes 1 month after DMBA treatment (Marchant, 1957; Marchant, 1959*b*; Mody, 1960; Kuwahara, 1967) is due to a direct and immediate destroying effect of DMBA on the small oocytes. After oral administration of DMBA the number of developing oocytes in follicles is secondarily reduced, while after intraperitoneal injection some follicles are directly destroyed in addition (Krarup, 1969*b*). The immediate effect of DMBA on the granulosa cells of the follicles, however, is a transient one, which causes a temporary acceleration of the follicle growth rate; when this stimulating effect has subsided the remaining follicles continue to develop normally (Pedersen and Krarup, 1969). The labelling index of the last remaining follicles was normal and their ability to ovulate was preserved (Fig. 4). Despite the severely reduced germ cell population and advanced preneoplastic changes, the remaining oocytes which ovulate can be fertilized and produce young (Krarup, not yet published).

Concurrently with the process of germ cell elimination pathological changes develop in the ovaries. It has been suggested that the neoplastic development is secondary to the premature elimination of oocytes and not caused by the carcinogen itself (Krarup, 1969*a*). This is supported by the observations that ovarian tumours invariably develop following genetic deletion of germ cells (Russell and Fekete, 1958; Murphy and Russell, 1963) and that, among four strains of mice, spontaneous ovarian tumours only occurred in that particular strain whose ovaries were physiologically exhausted of oocytes within the lifespan of the animals (Jones and Krohn, 1961). That the loss of oocytes and follicles after X-irradiation " was correlated with the onset of active growth " was noted by Guthrie (1958),

FIG. 21.—The bilateral ovarian histology and incidence of tumours at different ages after oral and intraperitoneal DMBA administration at 4 months. The mice are classified by the more advanced of the two ovaries according to a rising sequence of preference: potential preneoplasia—luteoma—microscopic granulosa cell tumour—macroscopic granulosa cell tumour—starting from the left on the figures. This dominant ovary is given in the left half of the double columns, while the less advanced of the two ovaries is indicated in the right half of the double columns. (1) Bilateral leukemic infiltration, (2) stroma cell tumour.

who found " the growth of the interstitial mass restrained in the presence of a residual follicle ".

The present data confirm that the neoplastic ovarian development is correlated with the elimination of germ cells. In 3-month-old animals the total number of oocytes after feeding of DMBA on day 21 varied between 289 and 5 (Krarup, 1969*b*); the preneoplastic changes were less advanced in the presence of many oocytes than when only few oocytes had survived, and Mic. G. had developed only in those two mice whose ovaries were nearly depleted of oocytes. Furthermore, the fact that the tumour development occurred faster after intraperitoneal injection of DMBA than after oral ingestion (Fig. 22) can be correlated with a faster destruction of oocytes in the intraperitoneally treated animals. Also the shorter tumour latency period in mice treated orally at 4 months than on day 21 (Fig. 21 and 22) may be explained by the fact that depletion of oocytes occurred faster in the 4-month-old animals (Krarup, 1969*b*). The delaying effect of surviving oocytes with intact follicles on tumorigenesis is probably a phenomenon similar to inhibition of tumour formation in the presence of a normal ovary after DMBA induction (Marchant, 1960), X-irradiation (Lick *et al.*, 1949; Kaplan, 1951) and intrasplenic ovary transplantation (Biskind and Biskind, 1948; Li and Gardner, 1949). It is most likely due to the production of steroids by surviving follicles.

### *Histogenesis of ovarian tumours*

Besides degeneration of oocytes, the early post-treatment changes include the appearance of empty rings and pseudofollicles. These characteristic structures have been noted by several authors after X-irradiation and described as " anovular follicles ". Their origin is unclear and had been ascribed to (a) remnants of small follicles in which the oocytes have degenerated, (b) differentiation of embryonal cells lying dormant in the ovarian stroma or (c) formation from the germinal (surface) epithelium (reviewed by Thung *et al.*, 1956). In the present study empty rings and pseudofollicles have not been observed to be connected with the surface epithelium. That they are formed from follicles whose oocytes have degenerated is unlikely, because oocytes in follicles which have a size comparable to pseudofollicles (*i.e.* type 3b and type 4 follicles; Pedersen and Peters, 1968) are not destroyed by DMBA. Their number decreases because the pool of small oocytes—from which they are recruited—is reduced (Krarup, 1969*b*; Krarup *et al.*, 1969). Furthermore, there is a considerable discrepancy between the number of empty rings and pseudofollicles present in a preneoplastic ovary and the number of type 3b and type 4 follicles in a normal ovary. In one preneoplastic ovary (Fig. 2) the total number of empty rings and pseudofollicles was roughly estimated to 1500 (counted on every tenth section), while the number of type 3b and type 4 follicles in a normal ovary is limited to less than 300 (Krarup, 1969*b*). It is therefore most likely, that empty rings and pseudofollicles have been formed from cells belonging to ovarian stroma. In the immature mouse ovary, follicle cells are known to derive from stroma cells (Peters and Pedersen, 1967) and it is possible that such cells may differentiate to follicle-like structures under these experimental conditions where oocytes are absent.

The potential preneoplastic changes, mainly characterized by diffuse luteinization, have been described previously (Marchant, 1957; 1959*b*). This stage

FIG. 22.—The bilateral ovarian histology and incidence of tumours at different ages after oral and intraperitoneal DMBA administration on day 21. For further explanation see Fig. 21. (1) Total degeneration, (2) bilateral leukemic infiltration, (3) haemorrhagic cyst, (4) stroma cell tumour.

represents the end point of the initiation phase; its further development into tumour occurs only in the presence of the pituitary (promoting factor) (Marchant, 1961*a*).

The cellular components from which the tumour originates has been a matter of discussion. According to Marchant the preneoplastic lutein tissue again disappears and early tumours are considered to arise from remnants of follicles (Marchant, 1957; Kuwahara, 1967), but such an origin cannot be the explanation for late tumours (Marchant, 1959*b*). Luteinized tissue coexisting with granulosa cell tumour was interpreted as a matter of maturation (Marchant, 1959*a*), and the possibility of granulosa cell tumour arising in a pre-existing luteoma was denied (Marchant, 1959*b*). Biancifiori *et al.* (1961), on the other hand, found it possible that luteoma and granulosa cell tumour represented two phases of growth of the same tumour, and Mody (1960) described foci of granulosa cells arising in aggregates of thecal luteinization.

The results of the present study seem to express the following developmental sequence. The stage of potential preneoplasia has a central position in the process. It is well defined by its diffusely luteinized tissue, derived from confluent corpora lutea and luteinized stroma, and its peripheral collection of pseudofollicles (Fig. 2). It develops during the process of oocyte elimination simultaneously in the two ovaries resulting in a bilateral stage of preneoplasia (Fig. 22). It occurs with a high incidence in the early phase of development and becomes progressively rarer as tumours develop.

That tumours develop from cells present in preneoplastic ovaries is suggested autoradiographically by the fact that in luteinized tissue foci of cells had incorporated $^{3}$HTdR, *i.e.* proliferated (Fig. 18). It is likely that the luteinized cells by proliferation give rise to undifferentiated (granulosa-like) daughter cells, which continue their proliferation and form the granulosa cell tumour. The same type of proliferation has been described for the intrasplenic rat ovary by Myhre (1962). In the intraperitoneally treated mice no follicles were present in preneoplastic ovaries. In these ovaries luteinized cells therefore seem to be the only possible source of the Mic. G. (Fig. 15). However, after DMBA by mouth early Mic. G. developed in preneoplastic ovaries that sometimes contained a residual follicle (Fig. 9), and the possibility of abnormal growth from surviving follicles therefore cannot be ruled out. Empty rings and pseudofollicles disappear slowly as tumours develop, but persist as a broad rim in atrophic ovaries (Fig. 5). It is therefore unlikely that they give rise to granulosa cell tumour. But the rare " stroma cell tumour " (Fig. 17) which consists almost entirely of pseudofollicles may represent the result of further growth of these structures. Luteomata are composed of the same cellular components as preneoplastic ovaries but their lutein cells show proliferation and invasion. They can be considered the result of neoplastic transformation of the luteinized tissue of preneoplastic ovaries. Luteomata never become macroscopic and lutein cells were not found in the large tumours in the present study. Luteinization therefore cannot be considered as a matter of maturation. Late Mic. G. developed within pre-existing luteomata depleted of oocytes and follicles with granulosa-like cells found diffusely among the lutein cells (Fig. 10, 11, 12). The lutein cells showed incorporation of $^{3}$HTdR as well as mitotic activity. This would indicate that the late Mic. G. develop as the result of proliferation of luteoma cells (Myhre, 1962).

*The bilateral evolution giving rise to unilateral tumour*

Comparing the bilateral development, it is seen (Fig. 21 and 22) that preneoplasia occurred in both ovaries and that luteoma and Mic. G. occurred unilaterally as well as bilaterally. If unilateral, the ovary opposite to a luteoma was usually preneoplastic or atrophic, while the ovary contralateral to a Mic. G. could be luteoma, preneoplastic or atrophic. Mac. G. was practically always unilateral and opposite to an atrophic ovary. A possible sequence of this bilateral ovarian development is suggested schematically in Fig. 23, which demonstrates the multipotentiality of ovarian parenchyma to differentiate in several directions dependent on the character of the hormonal environment.

Fig. 23.—Scheme of the bilateral ovarian development leading to unilateral macroscopic granulosa cell tumour and contralateral atrophy. The sequence of bilateral stages of pathology is correlated to the hormonal status as indicated by the type of vaginal smears.

Bilaterally existing preneoplastic ovaries have three possibilities for further development: (1) Both of them progress to form a tumour (luteoma or Mic. G.); or one of them develops into a tumour while the other one (2) remains preneoplastic or (3) atrophies. When the Mic. G. develops further to form the Mac. G. the opposite ovary invariably becomes atrophic.

The reason for this ultimate unilateral development may be found in changes in the hormonal balance (Shisa and Nishizuka, 1968). The role of overstimulation by pituitary gonadotrophins for the development of the tumours has been described after X-irradiation (Mühlbock, 1953) and intrasplenic ovary transplantation (Biskind and Biskind, 1944; Biskind *et al.*, 1953; Lipschutz *et al.*, 1964).

In both situations, the ovary loses its ability to control the output of gonadotrophic hormones soon after the induction procedure. Hypophysectomy prevents tumorigenesis in the spleen-grafted ovary (Kullander, 1956) and also in the DMBA treated ovary (Marchant, 1961*a*). Administration of oestrogen prevents tumorigenesis in the intrasplenic ovary (Li and Gardner, 1949), as well as in the irradiated ovary (Gardner, 1950), due to its suppressing action on gonadotrophic hormones. A hormonal imbalance was found in most mice of the present study. Though cyclic, the oestrogen stimulation of the vagina in mice with preneoplastic ovaries, and often in mice with luteoma or Mic. G., was irregular with prolongation of dioestrus and mixed oestrus smears. Similar changes were described by Marchant (1957) before tumour development. This would indicate that as follicles disappear the ability of the ovaries to produce the oestrogen necessary to control the production and/or release of hypophyseal gonadotrophins is decreased. However, other animals with Mic. G. showed permanent oestrus, suggestive of a continuous oestrogen production by the tumour. This was especially marked in those mice with Mic. G., in whom the contralateral ovary was atrophic. Nearly all mice with Mac. G. and opposite atrophic ovaries showed signs of a continuous oestrogen production. It is therefore likely (Fig. 23) that a Mic. G.—developed from a preneoplastic ovary or a luteoma under pituitary overstimulation—at a certain time becomes autonomic and starts producing oestrogen, which via the feed-back mechanism suppresses the production and/or release of pituitary gonadotrophins. Thereby the opposite ovary (preneoplastic, luteoma, or not autonomous Mic. G.) loses its pituitary stimulation necessary for further development. It therefore atrophies, while the hormone producing granulosa cell tumour, now independent of the presence of the pituitary (Kullander, 1956), continues its growth.

### REFERENCES

Berenblum, I. and Shubik, P.—(1947) *Br. J. Cancer*, **1**, 383.
Biancifiori, C., Bonser, G. M. and Cashera, F.—(1961) *Br. J. Cancer*, **15**, 270.
Biskind, G. R., Bernstein, D. E. and Gospe, S. M.—(1953) *Cancer Res.*, **13**, 216.
Biskind, G. R. and Biskind, M. S.—(1948) *Science, N.Y.*, **108**, 137.
Biskind, M. S. and Biskind, G. R.—(1944) *Proc. Soc. exp. Biol. Med.*, **55**, 176.
Deane, H. W. and Fawcett, D. W.—(1952) *Anat. Rec.*, **113**, 239.
Engle, E. T.—(1946) *Cancer Res.*, **6**, 578.
Fekete, E.—(1946) *Cancer Res.*, **6**, 263.
Gardner, W. U.—(1950) *Proc. Soc. exp. Biol. Med.*, **75**, 434.
Guthrie, M. J.—(1957) *Cancer, N.Y.*, **10**, 190.—(1958) *Cancer, N.Y.*, **11**, 1226.
Howell, J. S., Marchant, J. and Orr, J. W.—(1954) *Br. J. Cancer*, **8**, 635.
Jones, E. C. and Krohn, P. L.—(1961) *J. Endocr.*, **21**, 469.
Jull, J. W., Streeter, D. J. and Sutherland, L.—(1966) *J. natn. Cancer Inst.*, **37**, 409.
Kaplan, H. S.—(1951) *J. natn. Cancer Inst.*, **11**, 125.
Krarup, T.—(1967) *Acta path. microbiol. scand.*, **70**, 241.—(1969*a*) *Int. J. Cancer*, **4**, 61.—(1969*b*) *J. Endocr.* (in press).
Krarup, T., Pedersen, T. and Faber, M.—(1969) *Nature, Lond.*, **224**, 187.
Kullander, S.—(1956) *Acta endocr., Copenh.*, **22**, Suppl. 27.
Kuwahara, I.—(1967) *Gann*, **58**, 253.
Li, M. H. and Gardner, W. U.—(1949) *Cancer Res.*, **9**, 35.
Lick, L., Kirschbaum, A. and Mixer, H.—(1949) *Cancer Res.*, **9**, 532.
Lipschutz, A., Cerisola, H. and Panasewich, V. I.—(1964) *Acta Un. int. Cancr.*, **20**, 1412.

MARCHANT, J.—(1957) *Br. J. Cancer*, **11**, 452.—(1959*a*) *Br. J. Cancer*, **13**, 306.—(1959*b*) *Br. J. Cancer*, **13**, 652.—(1960) *Br. J. Cancer*, **14**, 514.—(1961*a*) *Br. J. Cancer*, **15**, 821.—(1961*b*) In 'The Morphological Precursors of Cancer'. University of Perugia, p. 709.—(1967) In 'The Prevention of Cancer'. Edited by Ronald W. Raven and Francis J. C. Roe. London (Butterworths), p. 269.
MODY, J. K.—(1960) *Br. J. Cancer*, **14**, 256.
MÜHLBOCK, O.—(1953) *Acta endocr., Copenh.*, **12**, 105.
MURPHY, E. D.—(1966) In 'Biology of the Laboratory Mouse'. Edited by Earl L. Green. New York, (McGraw-Hill Book Company) p. 534.
MURPHY, E. D. AND RUSSELL, E. S.—(1963) *Acta Un. int. Cancr.*, **19**, 779.
MYHRE, E.—(1962) *Acta Un. int. Cancr.*, **18**, 50.
PARKES, A. S.—(1926) *Proc. R. Soc.* B., **100**, 172.
PEDERSEN, T. AND KRARUP, T.—(1969) *Int. J. Cancer*, **4**, 495.
PEDERSEN, T. AND PETERS, H.—(1968) *J. Reprod. Fert.*, **17**, 555.
PETERS, H. AND PEDERSEN, T.—(1967) *Fert. Steril.*, **18**, 309.
RUSSELL, E. S. AND FEKETE, E.—(1958) *J. natn. Cancer Inst.*, **21**, 365.
SHISA, H. AND NISHIZUKA, Y.—(1968) *Br. J. Cancer*, **22**, 70.
THUNG, P. J., BOOT, L. M. AND MÜHLBOCK, O.—(1956) *Acta endocr., Copenh.*, **23**, 8.
ZYLICZ, E., SIPS, D., LEVY, E. AND PETERS, H.—(1967) *Acta cytol.*, **11**, 483.


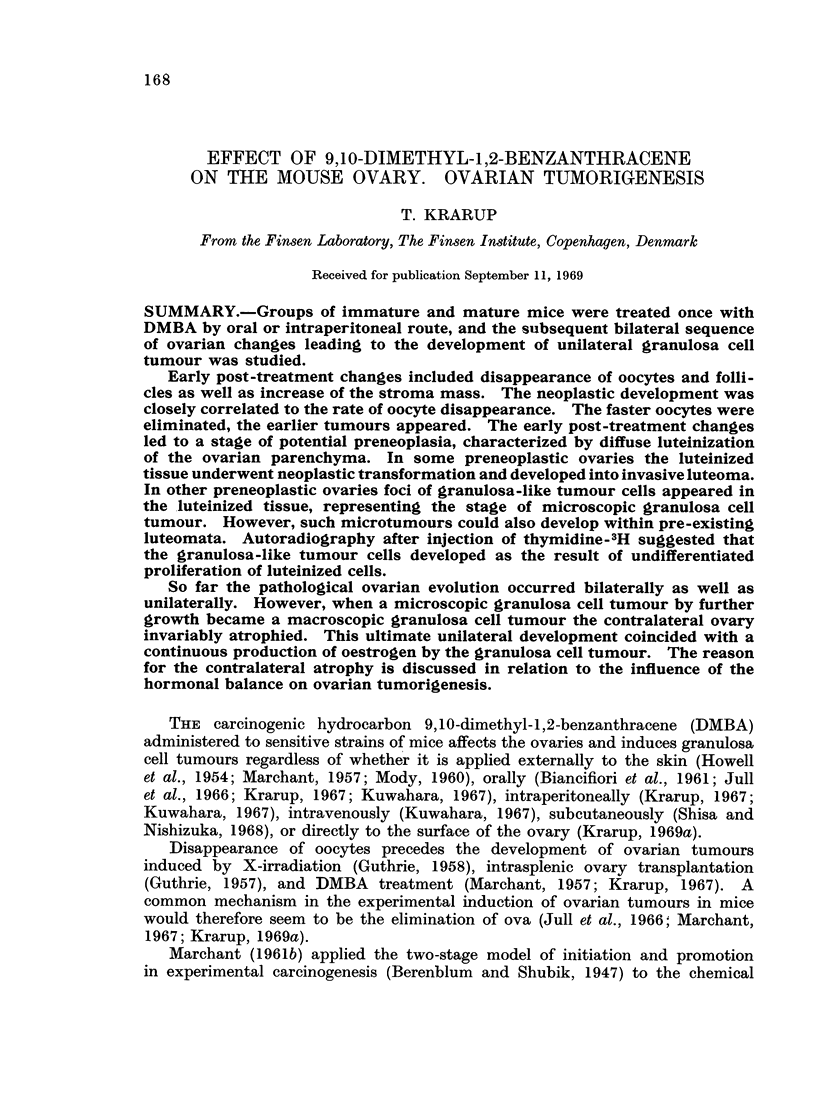

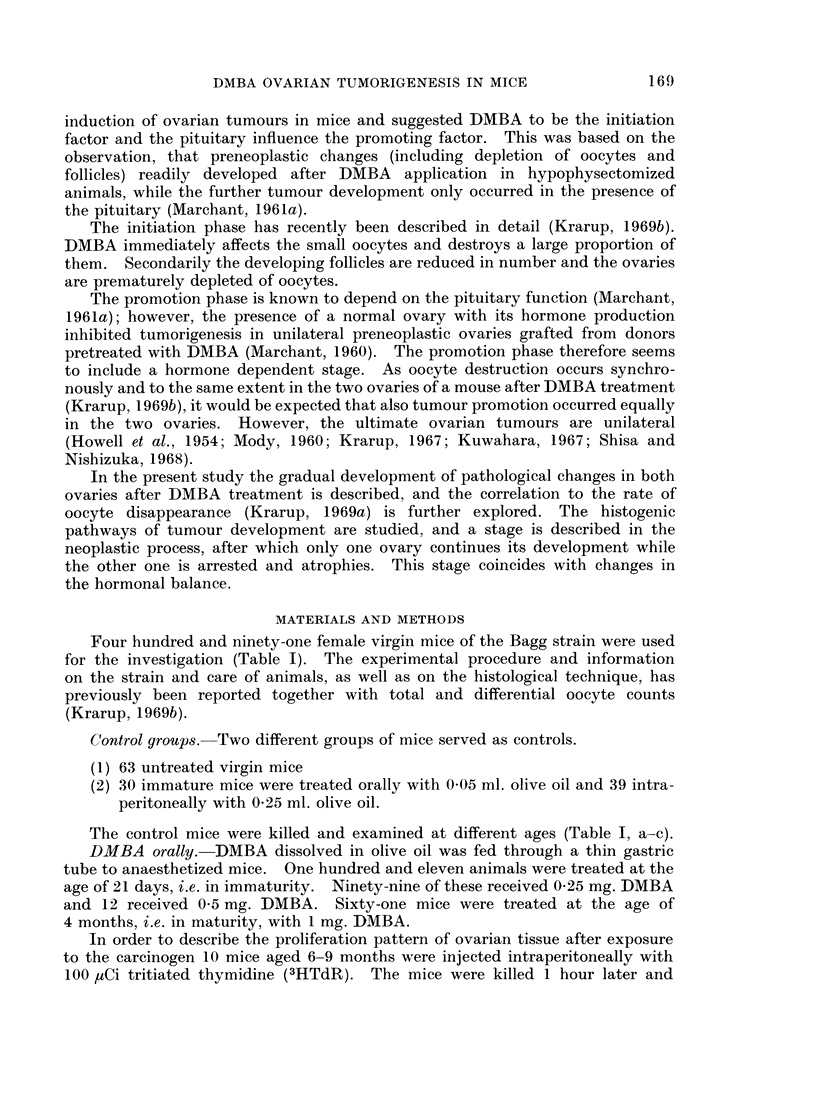

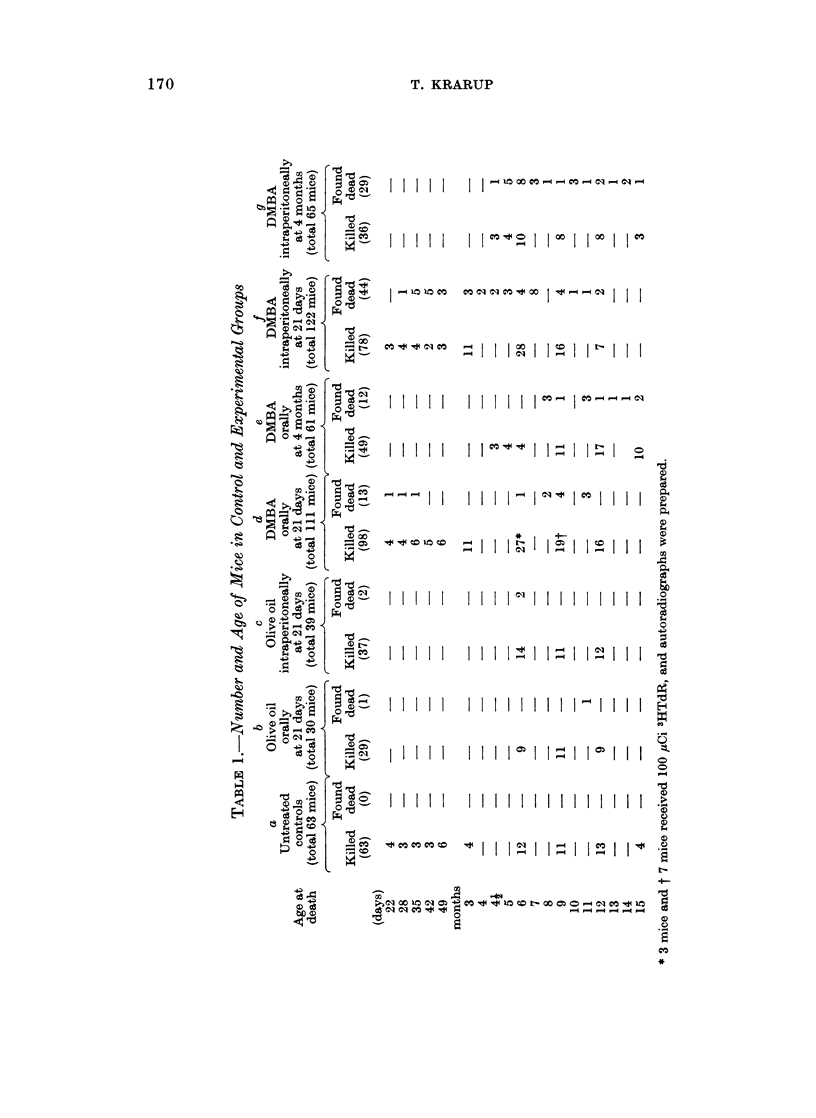

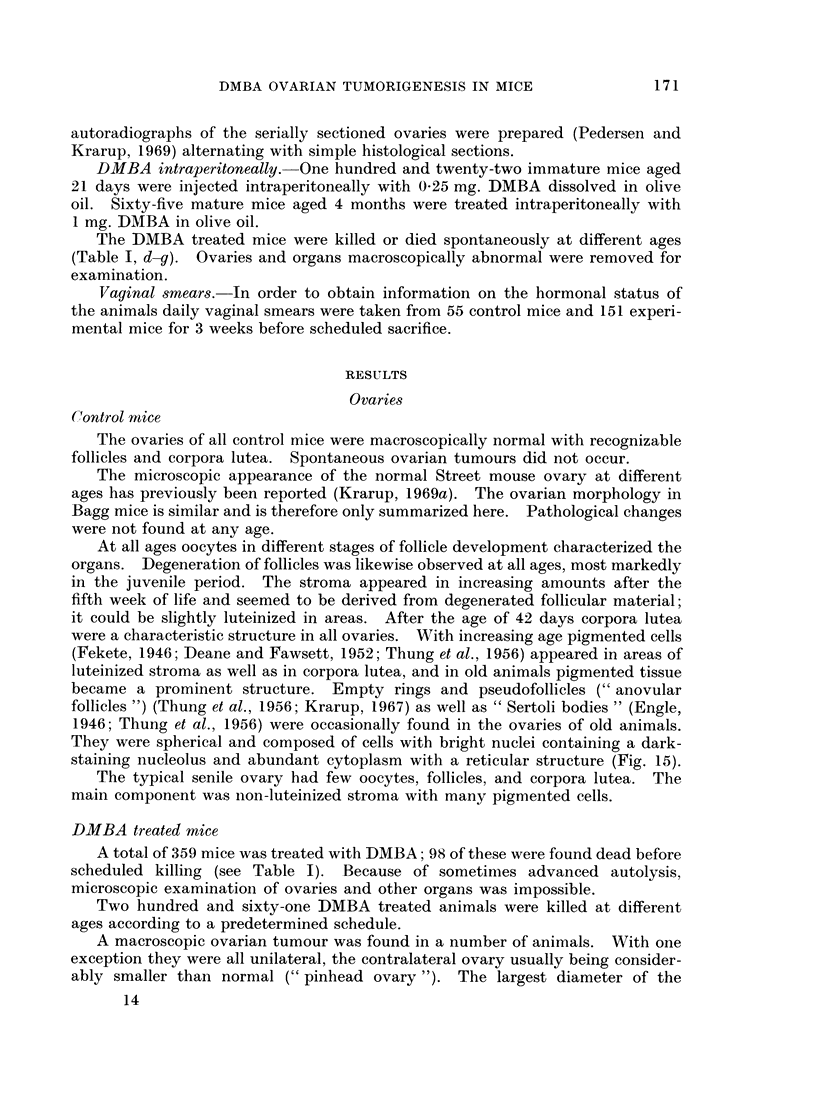

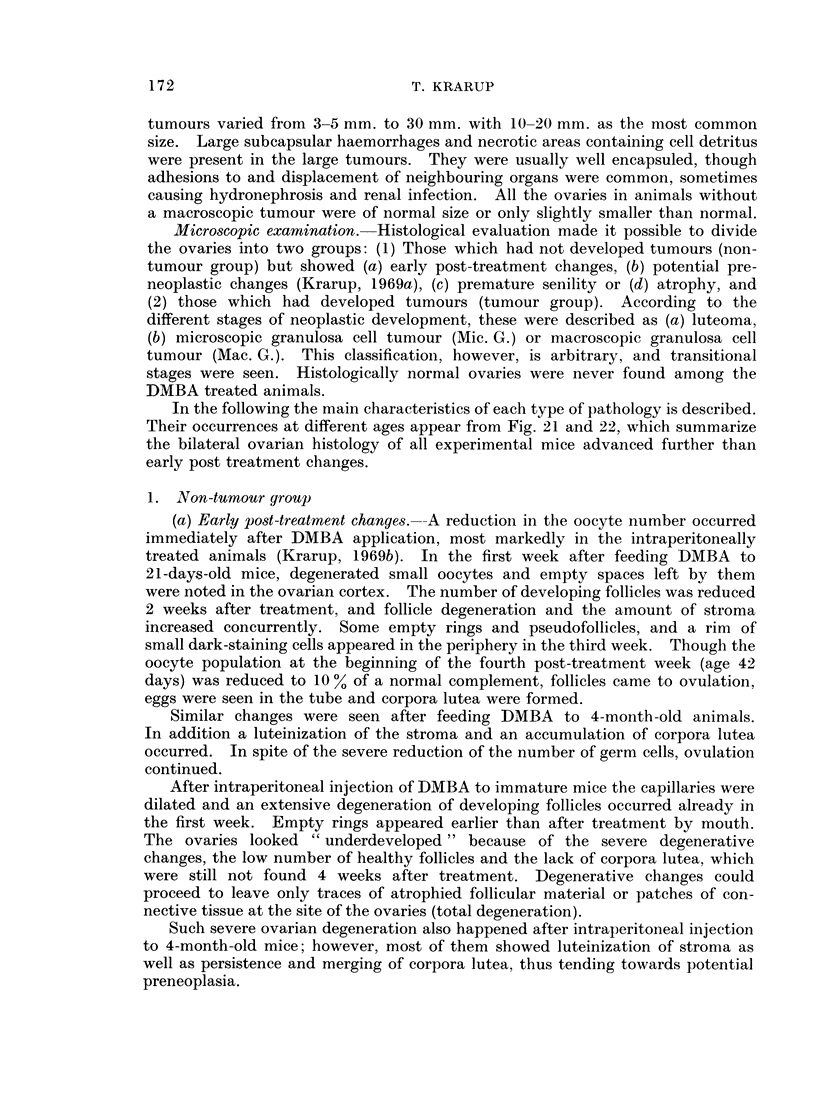

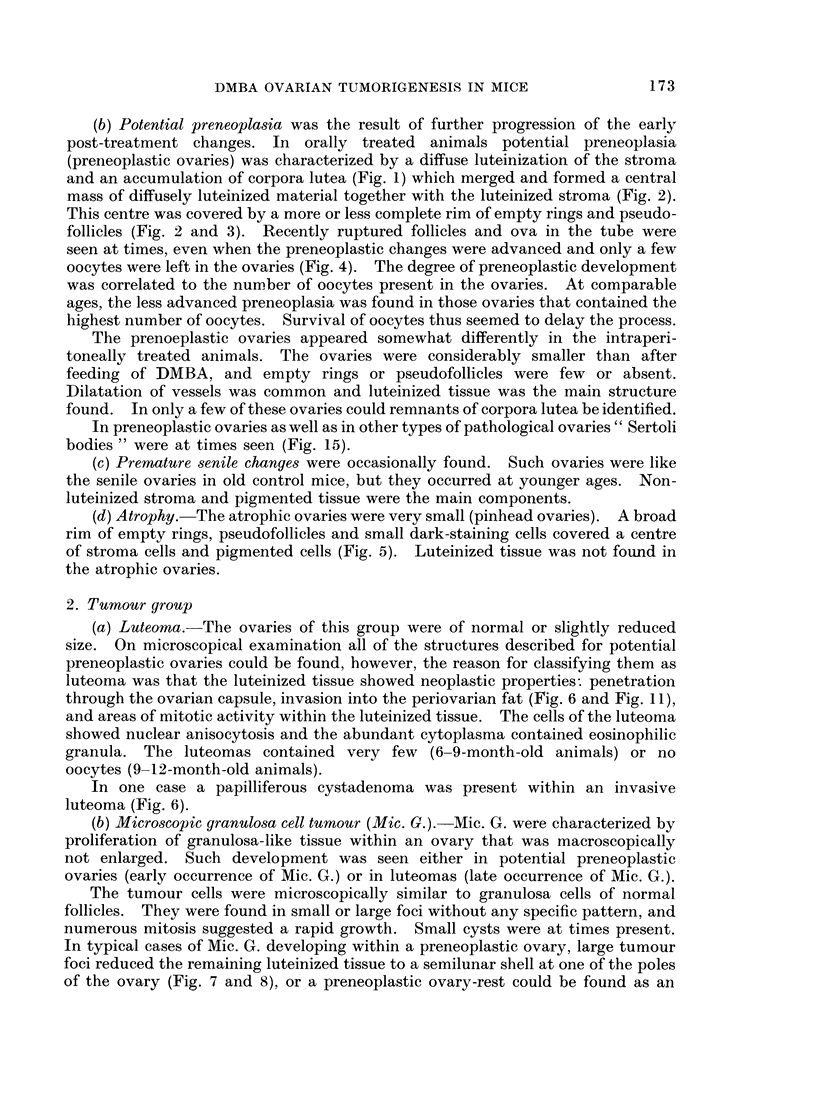

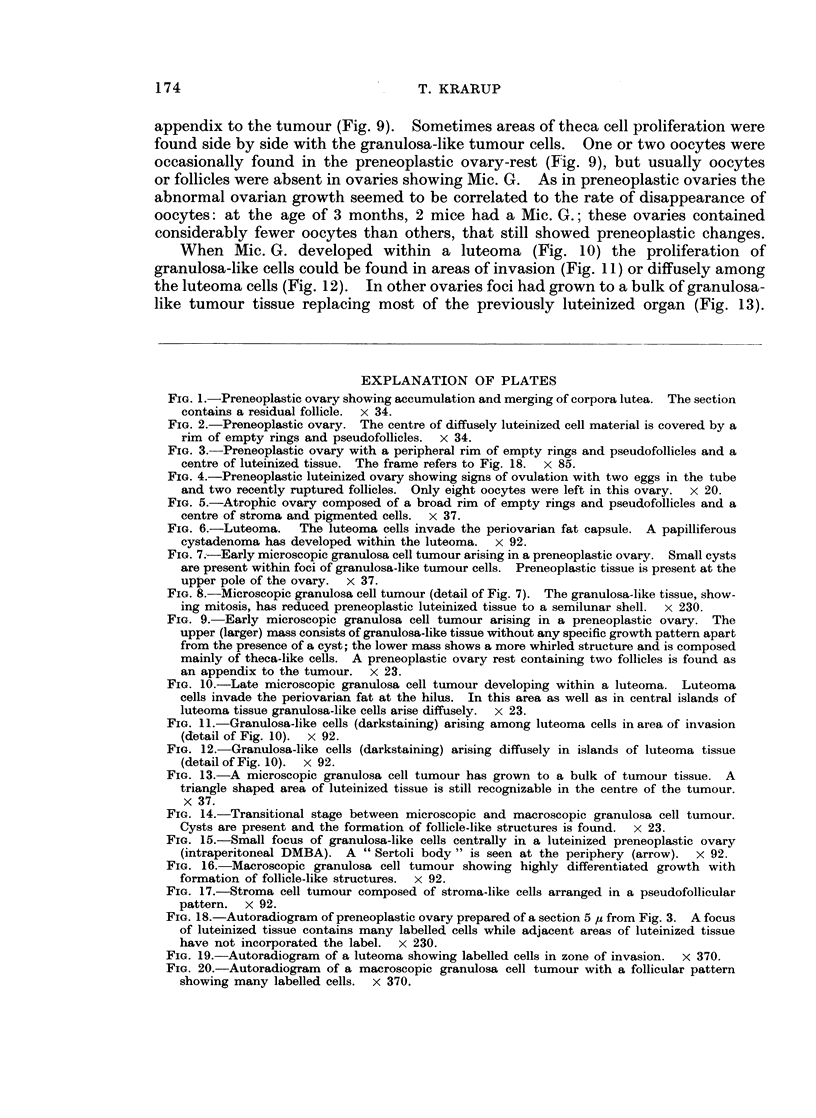

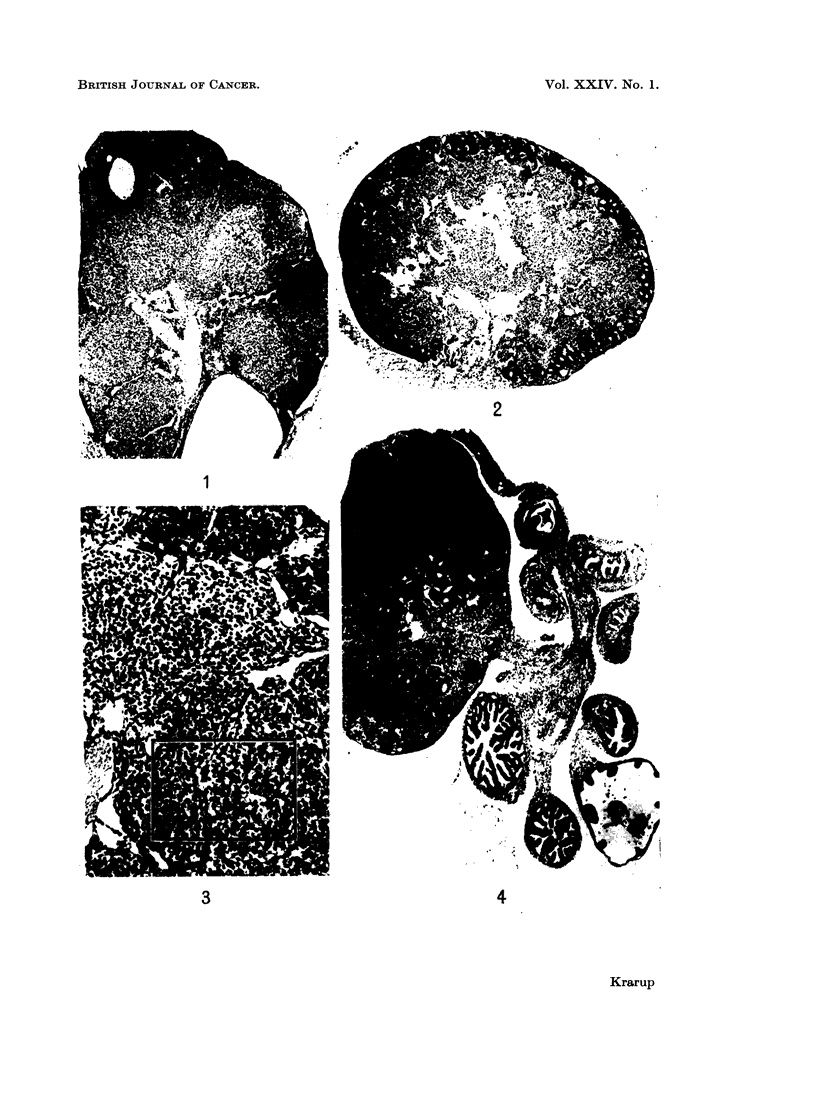

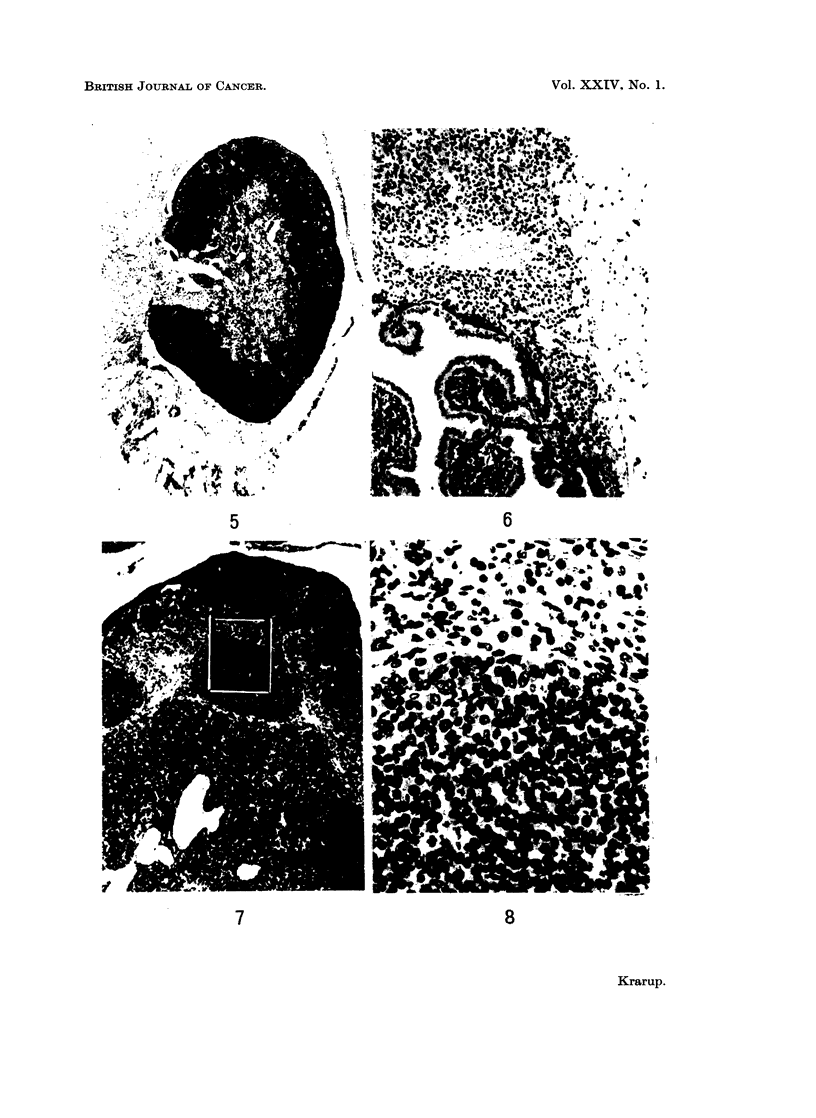

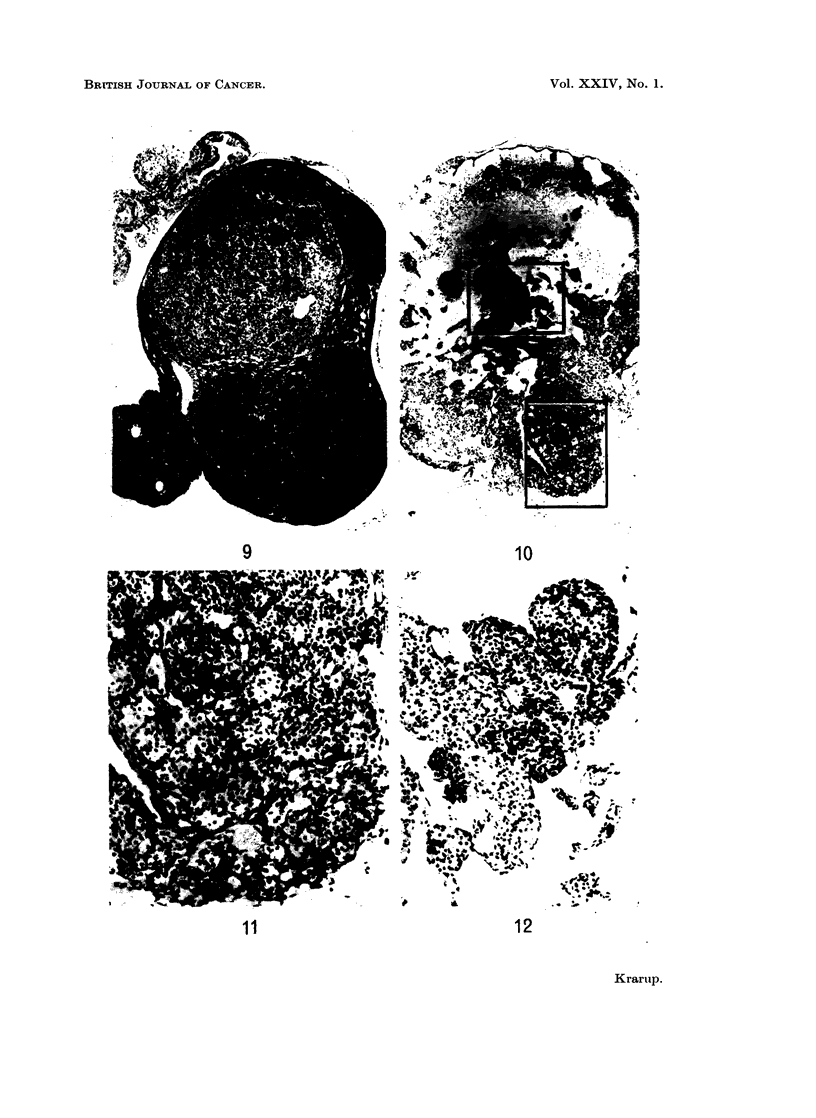

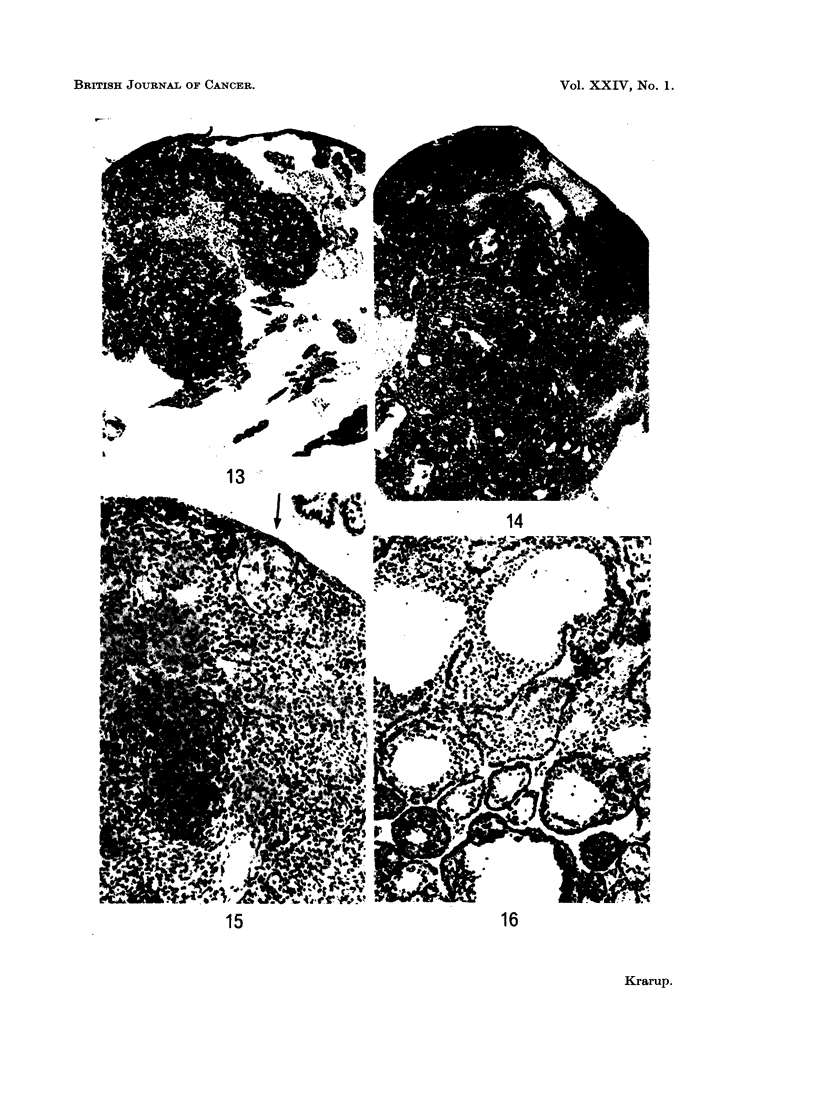

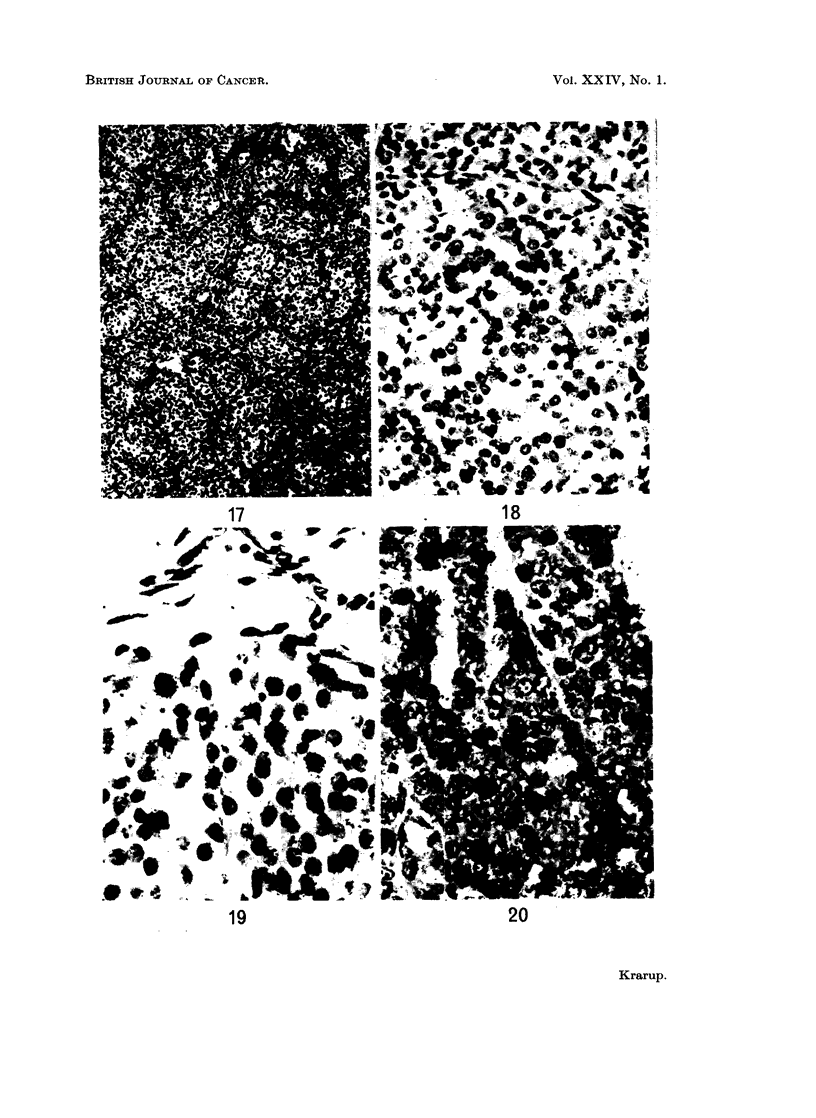

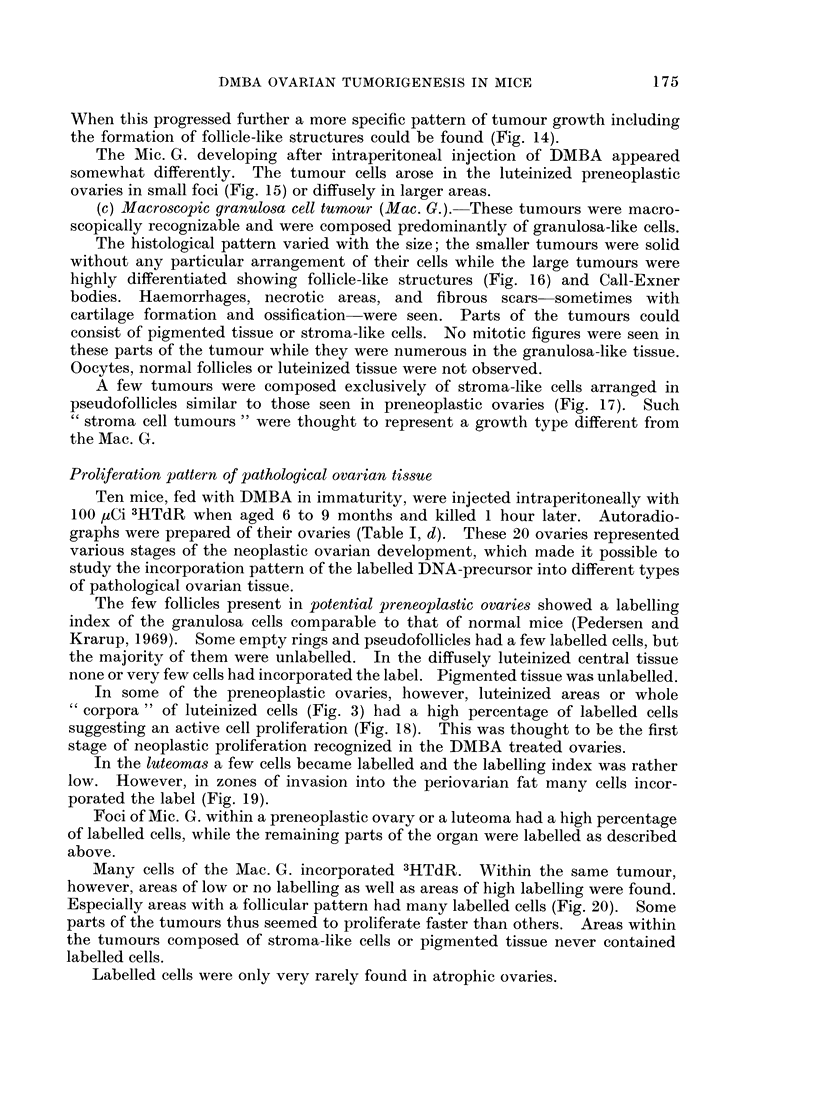

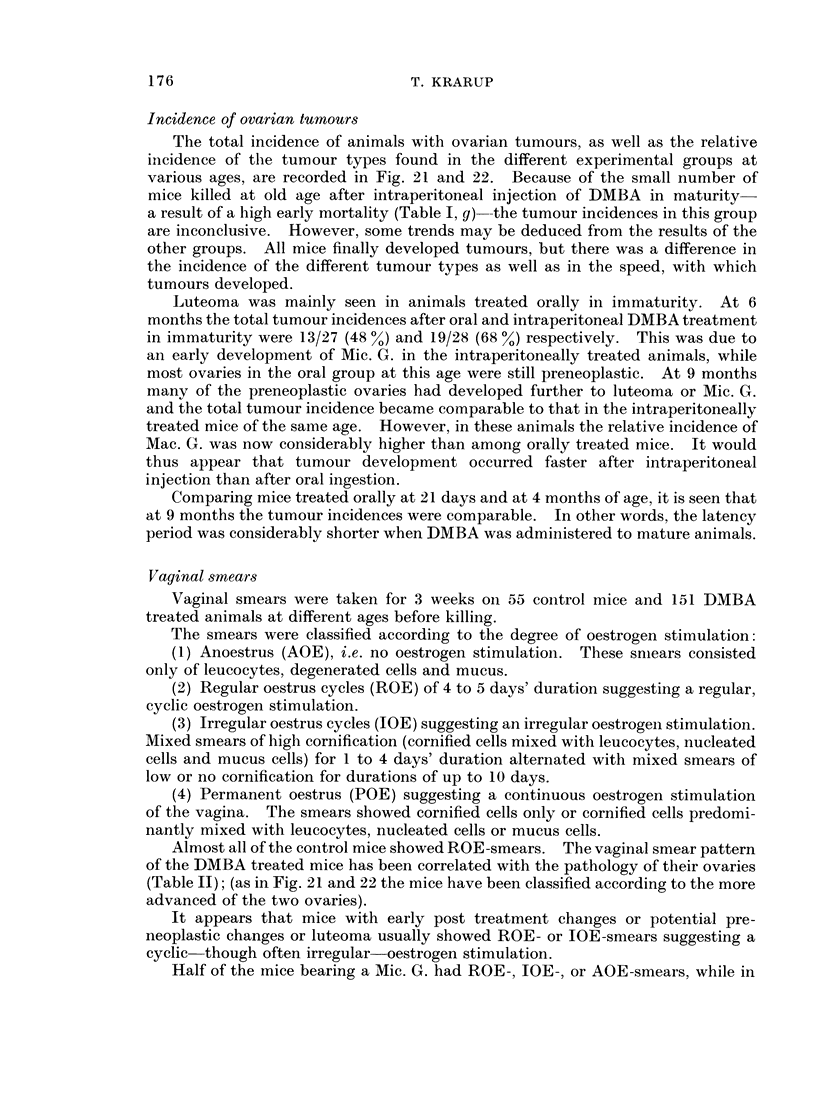

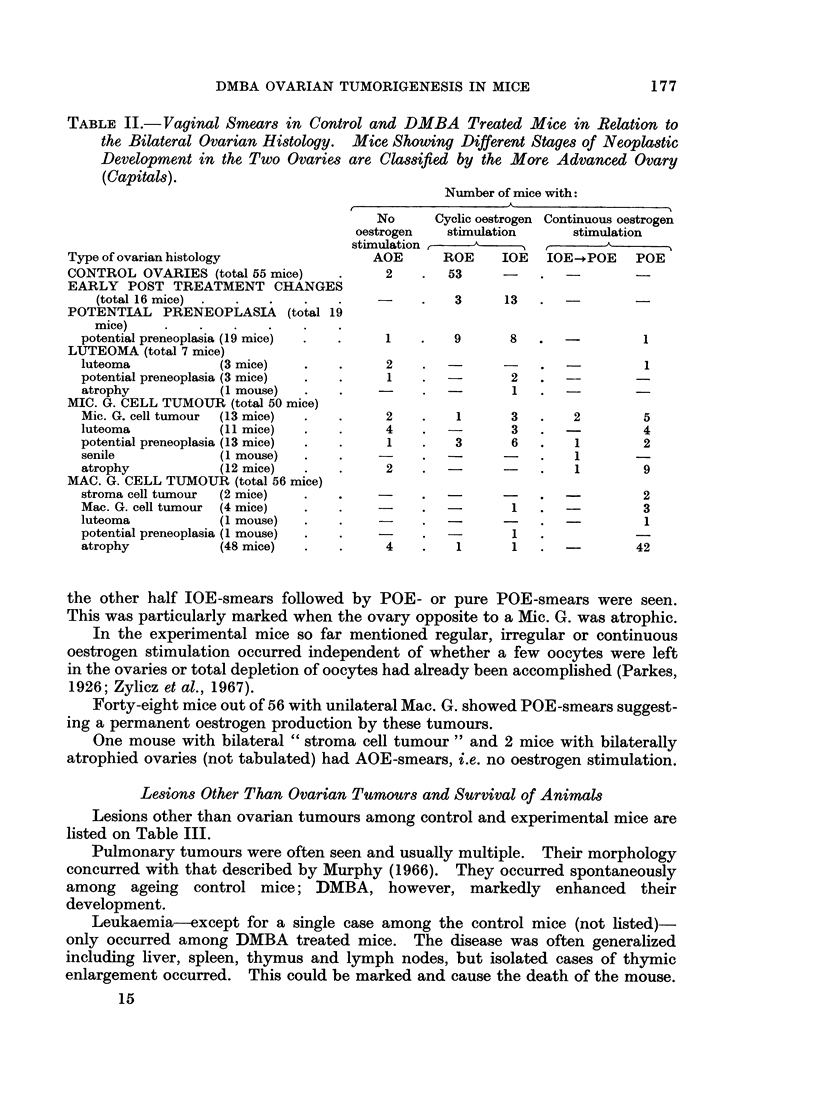

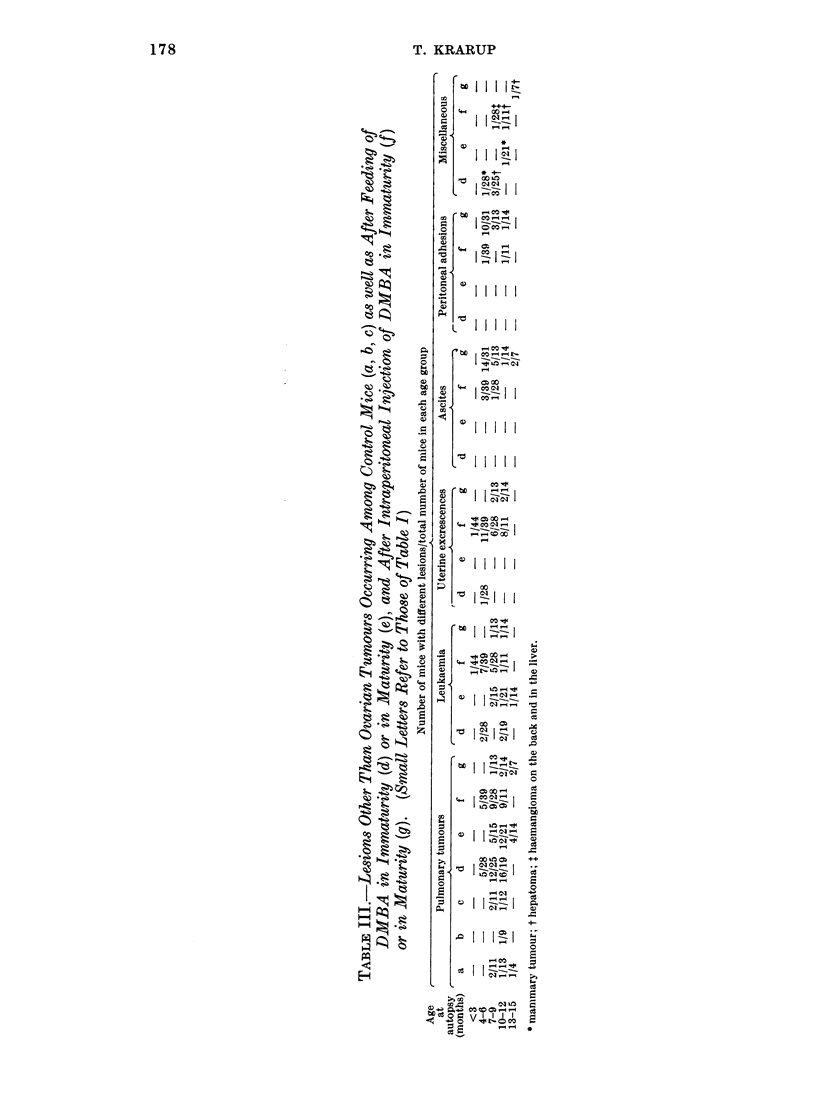

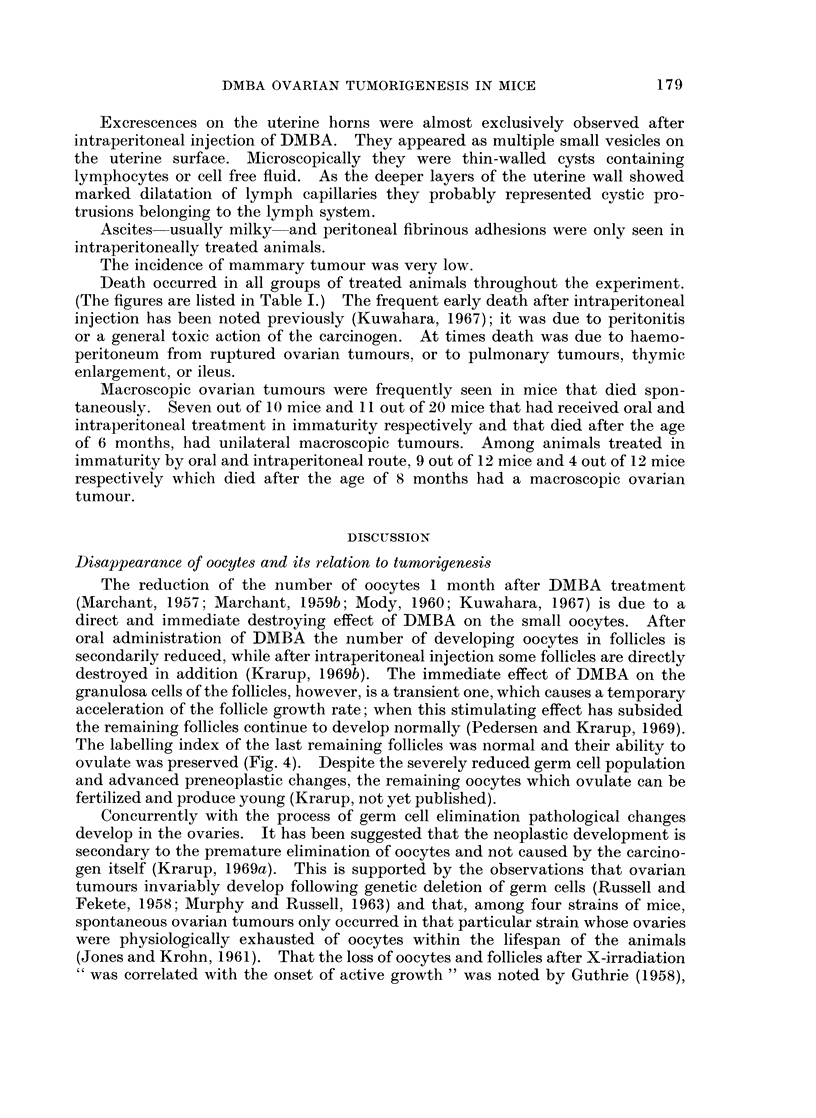

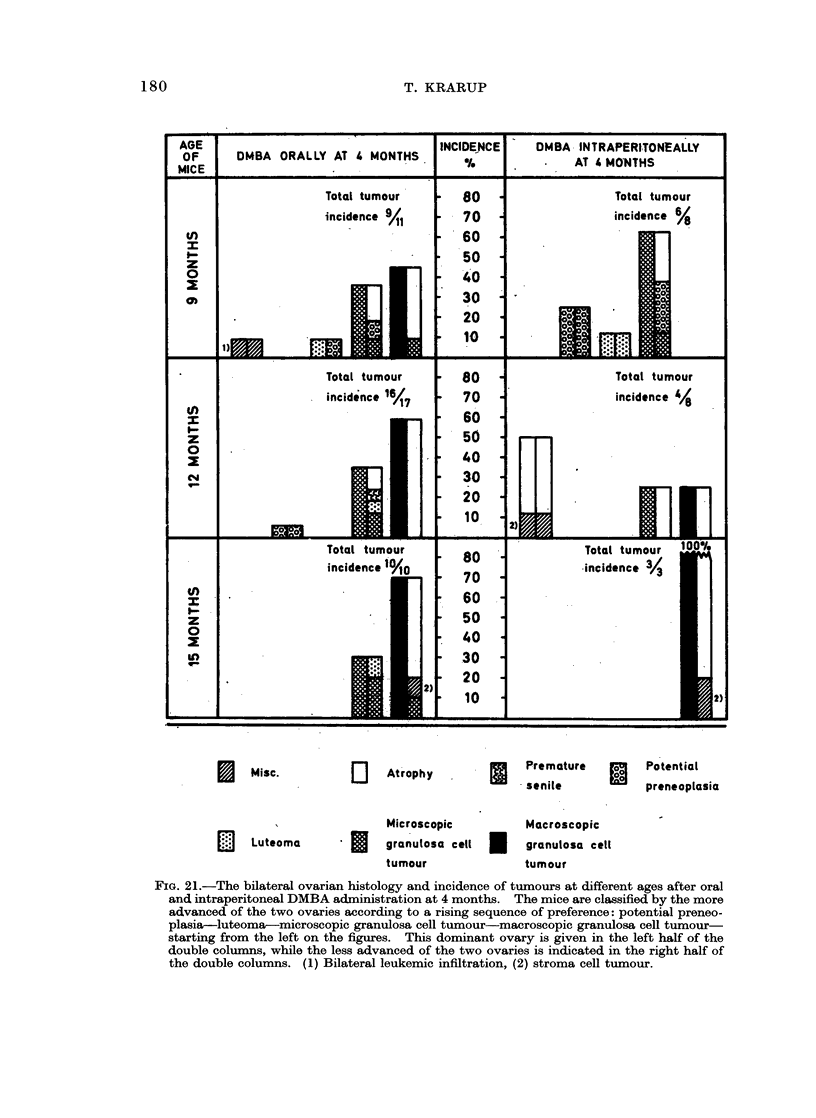

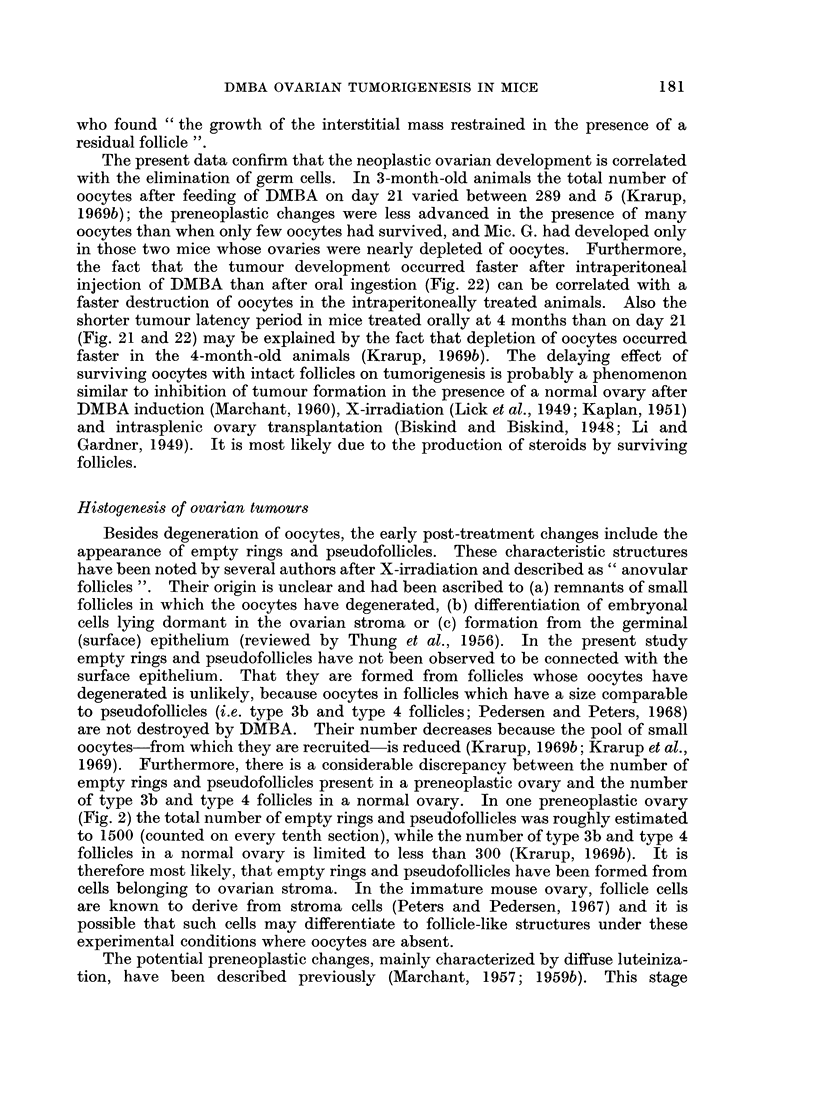

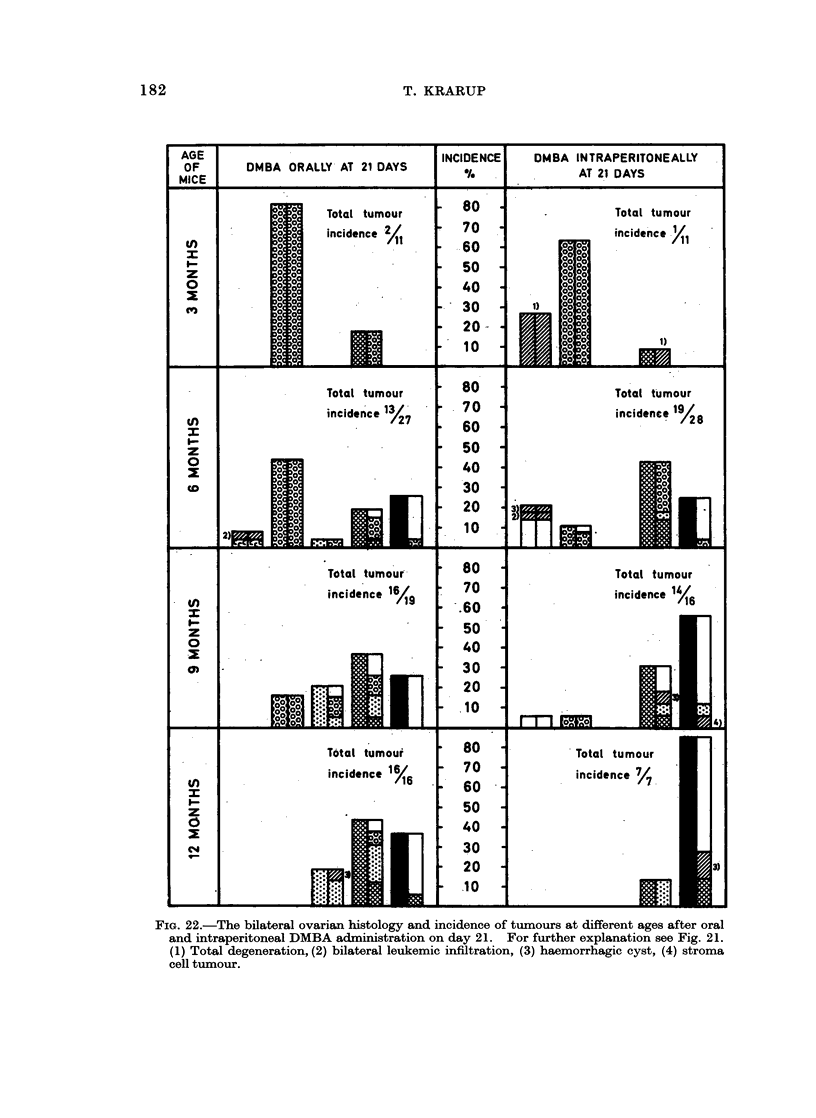

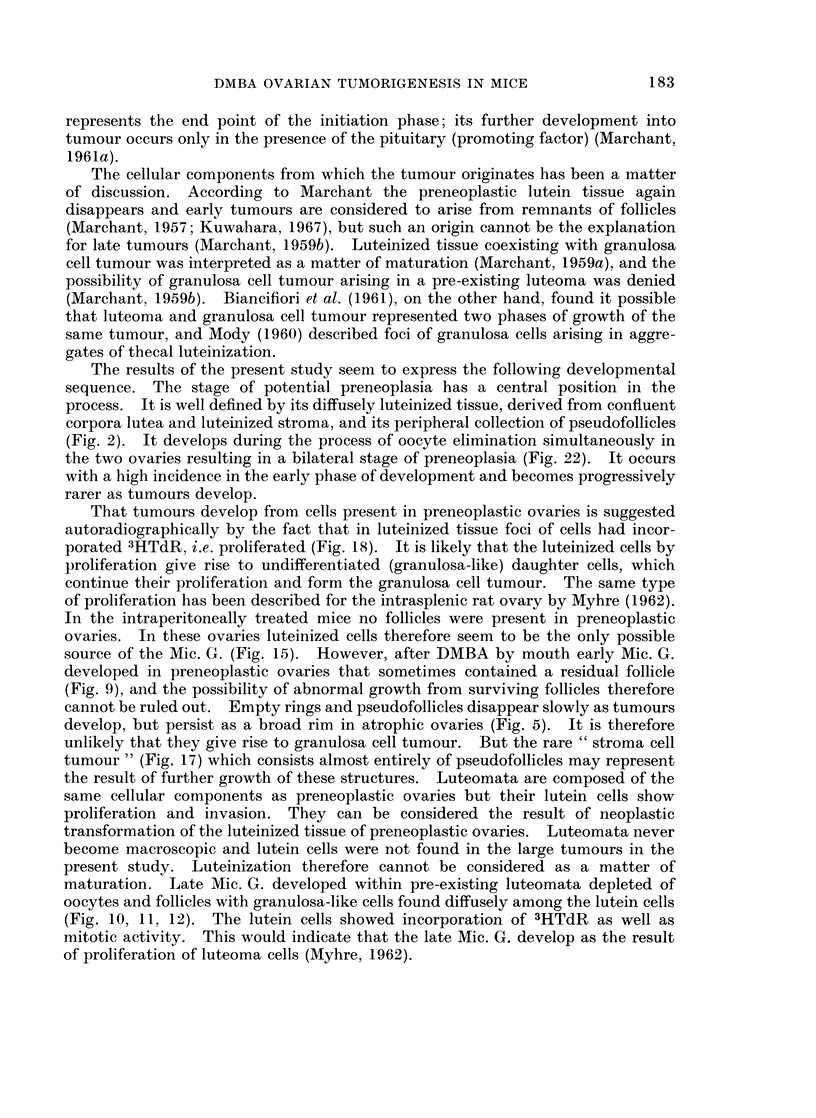

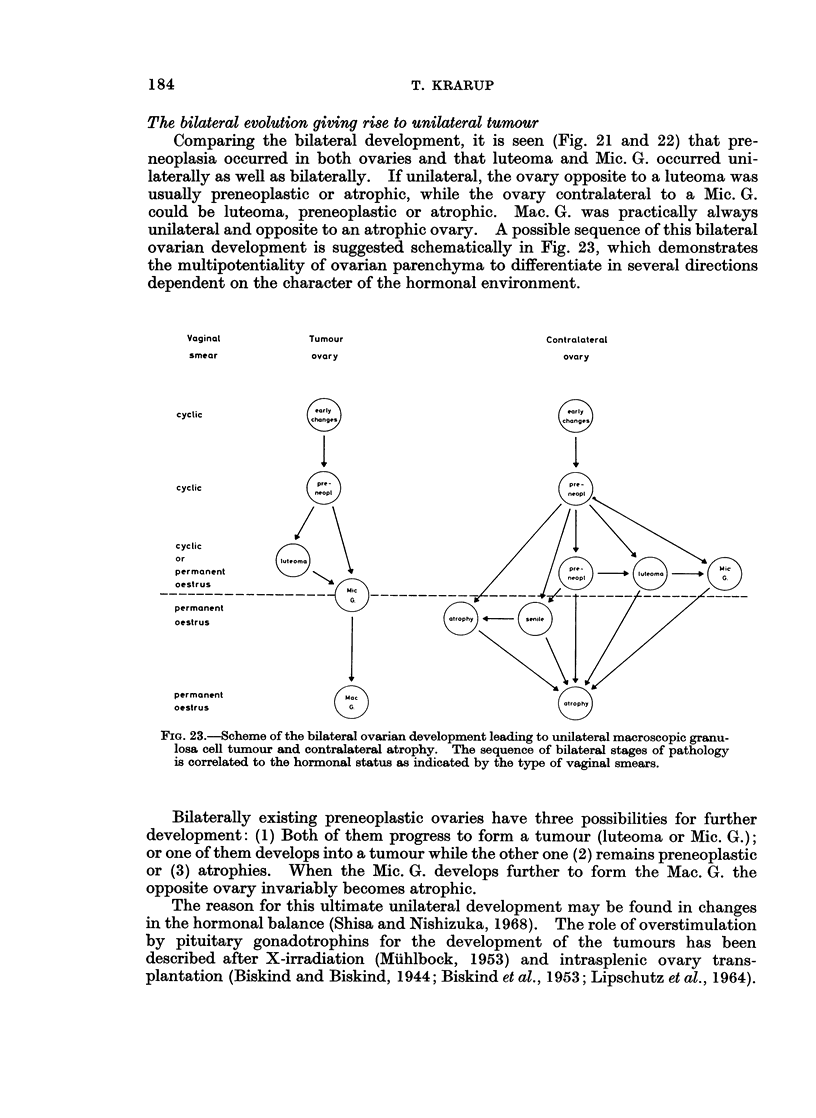

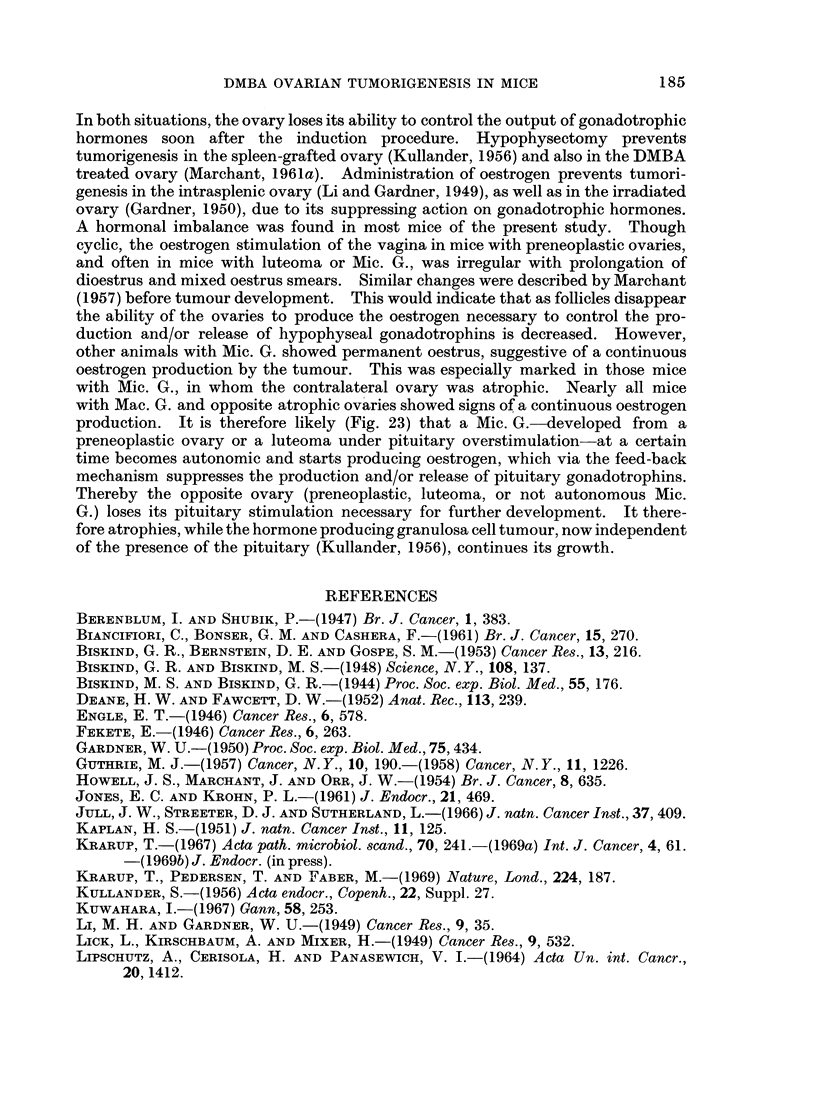

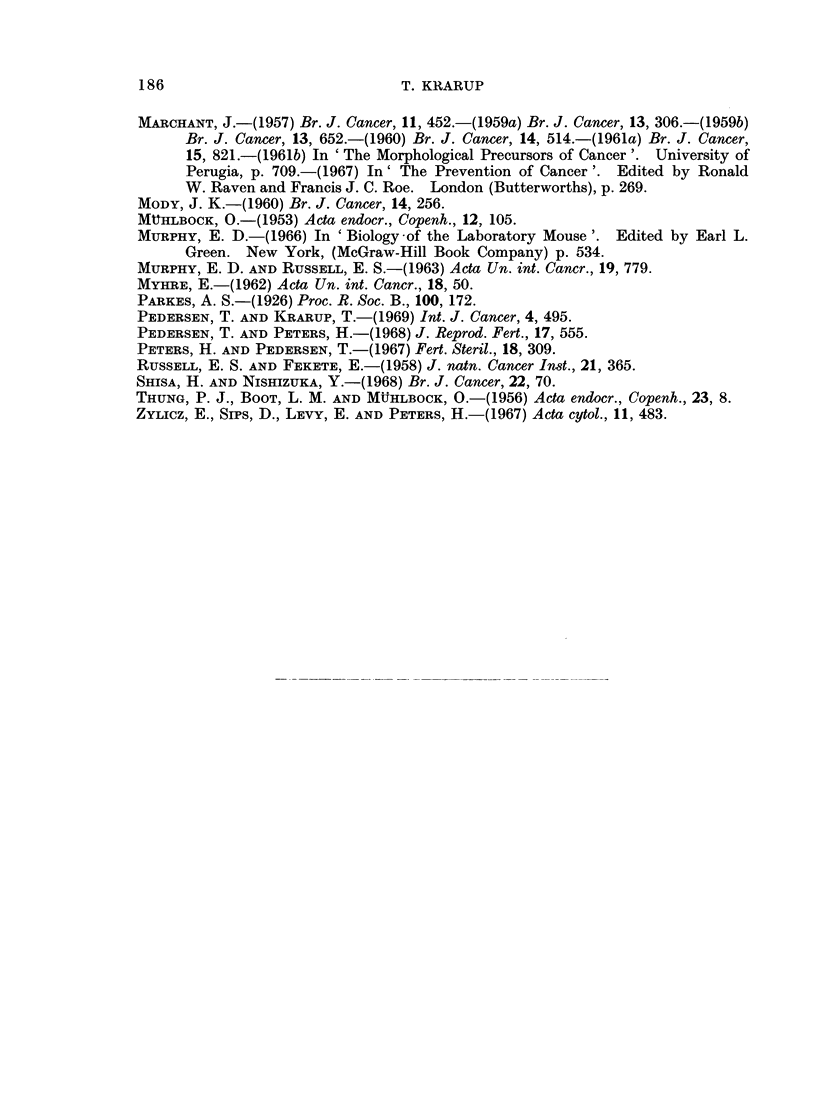


## References

[OCR_01369] Biskind G. R., Biskind M. S. (1948). Atrophy of Ovaries Transplanted to the Spleen in Unilaterally Castrated Rats; Proliferative Changes Following Subsequent Removal of Intact Ovary.. Science.

[OCR_01373] DEANE H. W., FAWCETT D. W. (1952). Pigmented interstitial cells showing brown degeneration in the ovaries of old mice.. Anat Rec.

[OCR_01379] HOWELL J. S., MARCHANT J., ORR J. W. (1954). The induction of ovarian tumours in mice with 9:10-dimethyl-1:2-benzanthracene.. Br J Cancer.

[OCR_01383] Jull J. W., Streeter D. J., Sutherland L. (1966). The mechanism of induction of ovarian tumors in the mouse by 7,12-dimethylbenz-[alpha]anthracene. I. Effect of steroid hormones and carcinogen concentration in vivo.. J Natl Cancer Inst.

[OCR_01385] Krarup T. (1967). 9:10-dimethyl-1:2-benzantracene induced ovarian tumours in mice.. Acta Pathol Microbiol Scand.

[OCR_01389] Krarup T., Pedersen T., Faber M. (1969). Regulation of oocyte growth in the mouse ovary.. Nature.

[OCR_01397] LIPSCHUTZ A., CERISOLA H., PANASEVICH V. I. (1964). THE ROLE OF PITUITARY HORMONES IN OVARIAN TUMOURIGENESIS.. Acta Unio Int Contra Cancrum.

[OCR_01405] MARCHANT J. (1960). The development of ovarian tumours in ovaries grafted from mice pretreated with dimethylbenzanthracene. Inhibition by the presence of normal ovarian tissue.. Br J Cancer.

[OCR_01411] MODY J. K. (1960). The actio of four carcinogenic hydrocarbons on the ovaries of IF mice and the histogenesis of induced tumours.. Br J Cancer.

[OCR_01417] MURPHY E. D., RUSSELL E. S. (1963). OVARIAN TUMORIGENESIS FOLLOWING GENIC DELETION OF GERM CELLS IN HYBRID MICE.. Acta Unio Int Contra Cancrum.

[OCR_01421] Pedersen T., Krarup T. (1969). Cell population kinetics in the mouse ovary after treatment with a chemical carcinogen (DMBA).. Int J Cancer.

[OCR_01423] Peters H., Pedersen T. (1967). Origin of follicle cells in the infant mouse ovary.. Fertil Steril.

[OCR_01426] RUSSELL E. S., FEKETE E. (1958). Analysis of W-series pleiotropism in the mouse: effect of WvWv substitution on definitive germ cells and on ovarian tumorigenesis.. J Natl Cancer Inst.

[OCR_01430] Zylicz E., Sips D., Levy E., Peters H. (1967). The vaginal smear in mice. A correlation of smear type and oocyte number.. Acta Cytol.

